# The heterologous expression of conserved *Glycine max* (soybean) *mitogen activated protein kinase 3* (*MAPK3*) paralogs suppresses *Meloidogyne incognita* parasitism in *Gossypium hirsutum* (upland cotton)

**DOI:** 10.1007/s11248-022-00312-y

**Published:** 2022-06-28

**Authors:** Vincent P. Klink, Nadim W. Alkharouf, Kathy S. Lawrence, Bisho R. Lawaju, Keshav Sharma, Prakash M. Niraula, Brant T. McNeece

**Affiliations:** 1 USDA ARS NEA BARC Molecular Plant Pathology Laboratory, Building 004 Room 122 BARC-West, 10300 Baltimore Ave., Beltsville, MD 20705 USA; 2 Department of Computer and Information Sciences, Towson University, Towson, MD 21252 USA; 3 Department of Entomology and Plant Pathology, Auburn University, 209 Life Science Building, Auburn, AL 36849 USA; 4 Department of Biological Sciences, Mississippi State University, Mississippi State, MS 39762 USA; 5 Department of Biochemistry, Molecular Biology, Entomology and Plant Pathology, Mississippi State University, Mississippi State, MS 39762 USA; 6 Present Address: Center for Computational Sciences High Performance Computing Collaboratory, Mississippi State University, Mississippi State, MS 39762 USA; 7 Present Address: Department of Plant Pathology, North Dakota State University, 1402 Albrecht Blvd., Walster Hall 306, Fargo, ND 58102 USA; 8 Present Address: Cereal Disease Laboratory, 1551 Lindig Street, Saint Paul, MN 55108 USA; 9 Present Address: Department of Biological Sciences, Delaware State University, 1200 North Dupont Highway, Science Center 164, Dover, DE 19901 USA; 10 Present Address: Nutrien Ag Solutions, 737 Blaylock Road, Winterville, MS 38703 USA; 11 Department of Biochemistry, Molecular Biology, Entomology and Plant Pathology, Auburn University, 209 Life Science Building, Auburn, AL 36849 USA

**Keywords:** Plant parasitic nematode, Mitogen activated protein kinase (MAPK), Effector triggered immunity (ETI) pathogen associated molecular pattern (PAMP) triggered immunity (PTI), *Gossypium hirsutum*, Cotton, *Glycine max*, Soybean, Overexpression, RNA interference (RNAi), Gene Ontology

## Abstract

Two conserved *Glycine max* (soybean) *mitogen activated protein kinase 3* (*MAPK3*) paralogs function in defense to the parasitic soybean cyst nematode *Heterodera glycines*. Gene Ontology analyses of RNA seq data obtained from *MAPK3-1*-overexpressing (OE) and *MAPK3-2*-OE roots compared to their control, as well as *MAPK3-1*-RNA interference (RNAi) and *MAPK3-2*-RNAi compared to their control, hierarchically orders the induced and suppressed genes, strengthening the hypothesis that their heterologous expression in *Gossypium hirsutum* (upland cotton) would impair parasitism by the root knot nematode (RKN) *Meloidogyne incognita*. *MAPK3-1* expression (E) in *G. hirsutum* suppresses the production of *M. incognita* root galls, egg masses, and second stage juveniles (J2s) by 80.32%, 82.37%, and 88.21%, respectfully. Unexpectedly, egg number increases by 28.99% but J2s are inviable. *MAPK3-2*-E effects are identical, statistically. *MAPK3-1*-E and *MAPK3-2*-E decreases root mass 1.49-fold and 1.55-fold, respectively, as compared to the pRAP15-*ccd*B-E control. The reproductive factor (RF) of *M. incognita* for *G. hirsutum* roots expressing *MAPK3-1*-E or *MAPK3-2*-E decreases 60.39% and 50.46%, respectively, compared to controls. The results are consistent with upstream pathogen activated molecular pattern (PAMP) triggered immunity (PTI) and effector triggered immunity (ETI) functioning in defense to *H. glycines*. The experiments showcase the feasibility of employing *MAPK3*, through heterologous expression, to combat *M. incognita* parasitism, possibly overcoming impediments otherwise making *G*. *hirsutum*’s defense platform deficient. MAPK homologs are identified in other important crop species for future functional analyses.

**Supplementary Information:**

The online version contains supplementary material available at 10.1007/s11248-022-00312-y.

## Introduction

Plant defense processes function through the recognition of epitopes associated directly or indirectly with the offending pathogen, referred to as pathogen activated molecular patterns (PAMPs) (Janeway 1989; Medzhitov and Janeway 1997; Schmelz et al. 2009; Manosalva et al. 2015; Mélida et al. 2020). (PAMP (pattern) triggered immunity (PTI) occurs by pattern recognition receptor (PRR) perception of PAMPS, providing a basal level of resistance (Jones and Dangl 2006). PTI is affiliated with a second defense tier called effector triggered immunity (ETI) whose activation can lead to the sacrifice of plant cells (Jones and Dangl 2006). Notably, PTI and ETI cross communicate, influencing the activity of each other’s defense function so they are not mutually exclusive entities (Yi et al. 2015; Chen et al. 2017; McNeece et al. 2019; Liu et al. 2020; Yuan et al. 2021; Dongus and Parker 2021; Lang et al. 2021). ETI and PTI function through mitogen activated protein kinase (MAPK) signaling, leading to an output defense response (Flor 1971; Tamkun et al. 1986; Wei et al. 1992; Kunkel et al. 1993; Grant et al. 1995; Century et al. 1995, 1997; Li and Chory 1997; Shapiro and Zhang 2001; Jonak et al. 2002; MAPK Group 2002; Hazzalin and Mahadevan 2002; Mackey et al. 2002; Coppinger et al. 2004; Zipfel et al. 2004, 2006; Veronese et al. 2006; Day et al. 2006; Jones and Dangl 2006; Chinchilla et al. 2007; Boudsocq et al. 2010; Knepper et al. 2011; Liu et al. 2013, 2020; Sun et al. 2014; Manosalva et al. 2015; Ma et al. 2020; Lang et al. 2021; Dongus and Parker 2021; Klink et al. 2021a). Pathogen effectors are capable of interfering with the activity of some of these proteins (Century et al. 1995, 1997; Desikan et al. 1998; Mackey et al. 2002, 2003; Axtell and Staskawicz 2003; Belkhadir et al. 2004; Kim et al. 2005; Lee et al. 2007; McNeece et al. 2019). The results are consistent with observations made for parasitic nematodes (Pant et al. 2014; Aljaafri et al. 2017; McNeece et al. 2017, 2019; Klink et al. 2021a). The relationship of these processes to pathogenic nematodes has been reviewed (Kaloshian and Teixeira 2019; Sato et al. 2019).

Studies employing RNA isolated from *Glycine max* (soybean) root cells undergoing parasitism by the pathogenic nematode *Heterodera glycines* demonstrate the cells, while undergoing a defense process, are expressing various PTI and ETI components that also function in defense (Klink et al. 2007, 2009, 2010a, b, 2011, 2021a; Matsye et al. 2011; Pant et al. 2014; Aljaafri et al. 2017; McNeece et al. 2017; Lawaju et al. 2018). Transgenic experiments demonstrate general aspects of plant defense to parasitic nematodes are conserved in composition and function with those components that act against other pathogen types (Pant et al. 2014; Aljaafri et al. 2017; McNeece et al. 2019; Klink et al. 2021a). Furthermore, their overexpression leads to an increase in the relative transcript abundances of genes that function in the defense process while their RNAi decreases their relative transcript abundances (Pant et al. 2014; McNeece et al. 2017, 2019; Aljaafri et al. 2017; Klink et al. 2021a). For example, *MAPK3-1* (Glyma.U021800) overexpression increases the relative transcript abundances of the hemicellulose-modifying *xyloglucan endotransglycosylase-hydrolase 43*, (*XTH43*) (Glyma.17G065100), the dominant *Resistance to heterodera glycines 4* (*Rhg4*) *serine hydroxymethyltransferase-5* (*SHMT-5*) (Glyma.08G108900), *reticuline oxidase-40* (*RO-40*) (Glyma.15G132800), *galactinol synthase-3* (*GS-3*) (Glyma.19G227800), *MAPK3-2* (Glyma.12G073000), *NONRACE-SPECIFIC DISEASE RESISTANCE1* (*NDR1-1*) (Glyma.12G214100), and secreted *pathogenesis related 1–6* (*PR1-6*) (Glyma.15G062400) (McNeece et al. 2019). Related overexpression experiments of the other *MAPK3* paralog (*MAPK3-2*) leads to an increase in the relative transcript abundances of the proven defense genes *RO-40*, *NON EXPRESSOR OF PR1* (*NPR1*) co-transcriptional regulator *TGA2-1* (Glyma.10G296200), *SHMT-5*, *NPR1-2*, *MAPK3-1*, and *PR1-6*. In contrast, the RNAi of *MAPK3-1* and *MAPK3-2* leads to a decrease in the relative transcript abundances of these same genes, respectively, with the transgenic roots being accompanied by susceptibility to *H. glycines* (McNeece et al. 2019). Therefore, the 2 *G. max* MAPK3 paralogs regulate the relative transcript abundance of defense genes in common with each other as well as those that are uniquely expressed in relation to the gene activity of each *MAPK3* paralog. Other experiments have also demonstrated this point (Niraula et al. 2020a).

As a rapid way in identifying pathogen defense pathways, a related root transformation platform has been developed for *Gossypium hirsutum* (upland cotton) (Pant et al. 2015). The development of a *G. hirsutum* genetic transformation system has allowed for the examination of *G. max NPR1-2*, *NDR1-1*, *XTH43*, and an -hydroxynitrile glucosidase (*g-4*) (Glyma.11G129600), showing their heterologous expression suppresses *M. incognita* parasitism (Pant et al. 2015; 2016; McNeece et al. 2017; Niraula et al. 2020b; Klink et al. 2021a).

The analysis presented here examines the effect that the heterologous expression of the *G. max MAPK3-1* and *MAPK3-2* has on *M. incognita* parasitism of *G. hirsutum*, providing key information on an important defense signaling node. The expression leads to a significant decrease in *M. incognita* parasitism. The results are placed into context by providing a relationship of these results to previously reported observations.

## Materials and methods

### Bioinformatics

The *A. thaliana* proteome is used to obtain its 20 MAPK protein sequences, including MAPK3 (AT3G45640) (Arabidopsis Genome Initiative 2000). The *A. thaliana* MAPK protein sequences are used to extract the *G. max* MAPK3-1 and MAPK3-2 (MAPK3-1 and MAPK3-2) protein sequences from its housed proteome at from Phytozome (https://phytozome.jgi.doe.gov) through a Basic Local Alignment Search Tool program (BLAST) query (Altschul et al. 1990). The default settings, include Target type: Proteome; Program: BLASTP-protein query to protein database; Expect (e) threshold: -1; Comparison matrix: BLOcks SUbstitution Matrix 62 (BLOSUM62); Word (W) length: default = 3; number of alignments to show: 100 allowing for gaps and filter query, in order that they appear on the BLAST program. (Goodstein et al. 2012; McNeece et al. 2019). The MAPK3-1 and MAPK3-2 protein sequences are used in pairwise comparisons employing the EMBOSS Program Needle, Version 6.6.0 to compare MAPK3-1 and MAPK3-2 in the Matrix; EBLOSUM62; Gap open, 10.0; Gap extend, 0.5; End Gap Penalty, false; End Gap Open Penalty, 10.0; End Gap Extension Penalty, 0.5 (Henikoff and Henikoff, 1992). The MAPK3-1 and MAPK3-2 protein sequences are used in BLAST queries of the *G. hirsutum* proteome housed at Phytozome using the EMBOSS 001 EBLOSUM62 Matrix in default settings with a gap penalty of 10.0 and extend penalty of 0.5 to obtain their homologs (Henikoff and Henikoff, 1992; Goodstein et al. 2012). The conserved domain analyses for the MAPK3-1 and MAPK3-2 proteins are performed according to Lu et al. (2020) using Conserved Domain Database (CDD) v3.19 in default settings. Multiple protein sequence alignments are performed using Clustal Omega under default settings (Sievers and Higgins, 2014).

### Proteomes employed

The proteomes of *G. hirsutum, G. max, Manihot esculenta*, *Zea mays*, *Oryza sativa*, *Triticum aestivum*, *Hordeum vulgare*, S*orghum bicolor*, *Brassica rapa*, *Solanum tuberosum*, *Solanum lycopersicum*, and *Beta vulgaris*, each housed at Phytozome, are mined further for MAPK-like proteins, including MAPK3. The *Elaes* guineensis and *Saccharum officinalis* proteomes are mined for MAPK-like proteins, including MAPK3 and are housed at PalmXplore (https://palmxplore.mpob.gov.my/palmxplore/) and the Sugarcane Genome Hub (https://sugarcane-genome.cirad.fr/), respectfully (Singh et al. 2013; Garsemeur et al. 2018; Ong et al. 2020). (Goodstein et al. 2012; Singh et al. 2013; Garsemeur et al. 2018; Ong et al. 2020).

### RNA seq analyses

The RNA sequencing (RNA seq) data under examination in this analysis is obtained from Alshehri et al. (2018) BioProject ID PRJNA664992, Submission ID: SUB8182387. The RNA used as template in the RNA seq analyses had been isolated from the respective *MAPK* overexpression (*MAPK*-OE), *MAPK* RNA interference (RNAi) (*MAPK*-RNAi), and respective OE (pRAP15-*ccd*B plasmid), and RNAi (pRAP17-*ccd*B plasmid) controls. Single replicate generation of RNA seq data, confirmed by RT-qPCR of the targeted genes, of RNA isolated from *MAPK3-1*-OE, *MAPK3-1*-RNAi, *MAPK3-2*-OE, *MAPK3-2*-RNAi and the pRAP15-*ccd*B (OE control) pRAP17-*ccd*B (RNAi control), are analyzed here (McNeece et al. 2019; Niraula et al. 2020b). The RT-qPCR-confirmed expression of genes identified in the RNA seq study and other genes (Sharma et al. 2020; Lawaju et al. 2020; Niraula et al. 2020b; Klink et al. 2021b). The accompanying Gene Ontology (GO) analyses are performed on the protein sequences composing the lists of induced and suppressed genes using PhytoMine (https://phytozome.jgi.doe.gov/phytomine/begin.do) (Goodstein et al. 2012). Graphs are generated using Excel.

### Plasmid details

The Gateway-compatible, 14,758 bp, pRAP15 plasmid expression (E) vector is used in the transgenic analysis of *G. hirsutum* (Matsye et al. 2012; Pant et al. 2015, 2016; McNeece et al. 2017; Niraula et al. 2020a; Klink et al. 2021a). A related plasmid, pRAP17 (15,596 bp, based off of the p*7GWIWG2(II) backbone) designed for RNA interference (RNAi) work but not used in the analysis has been generated (Karimi et al. 2002, 2007; Curtis and Grossniklaus 2003; Klink et al. 2009, 2021a). The pRAP15 plasmid is based off of the p*7WG2D vector, allowing the efficient directional cloning of genes at its attR recombination sites (Karimi et al. 2002, 2007; Curtis and Grossniklaus 2003; Matsye et al. 2012; Klink et al. 2021a). The pRAP15 plasmid, having the chloramphenicol-*ccd*B (Cm[r]-*ccd*B) gene (Invitrogen) (*ccd*B) that is lethal to *E. coli* TOP10 cells and acts as a selectable marker at the site where the candidate resistance gene (CRG) (i.e., *MAPK3-1* or *MAPK3-2*) would be engineered, also functions as the transgenesis control (pRAP15-*ccd*B) in gene expression, *M. incognita* parasitism and root mass experiments (Tam and Kline 1989; Bernard et al. 1991; Salmon et al. 1994; Karimi et al. 2002; Curtis and Grossniklaus 2003; Matsye et al. 2012; Klink et al. 2021a). Maintenance of the original, un-engineered, pRAP15 plasmid (lacking the insertion of a CRG transgene) is accomplished by the presence of the Cm(r)-*ccd*B lethality gene, selected using One Shot *ccd*B Survival 2 T1 R Competent Cells (Invitrogen) (Klink et al. 2021a). The tetracycline resistance gene (TetR), inserted outside of the left and right border, has been added during the development of pRAP15 to facilitate selection in *E. coli* or, importantly, *A. rhizogenes* or other bacteria. The *MAPK3-1* and *MAPK3-2* expression in *G. hirsutum* is driven by the figwort mosaic virus (FMV) sub-genomic transcript (Sgt) promoter consisting of a 301-bp FMV Sgt promoter fragment (sequence − 270 to + 31 from the transcription start site [TSS]) in pRAP15 (Bhattacharyya et al. 2002). The pRAP15 plasmid has been used to directionally clone the *G. max MAPK3-1* and *MAPK3-2* genes for overexpression in soybean but has been used here to obtain its heterologous expression in *G. hirsutum* (McNeece et al. 2019). The pRAP15 plasmid has 2 selectable reporters. The pRAP15 plasmid has the *enhanced green fluorescent protein* (e*GFP*) gene reporter for visual selection of transgenic plant tissue (Matsye et al. 2012; Klink et al. 2021a). The transcription of the e*GFP* gene is driven by the *rol*D promoter (Haseloff et al. 1997). The e*GFP* gene cassette is terminated by t35S translational terminator for effective visual reporting in plant tissue (White et al. 1985; Elmayan and Tepfer 1995; Haseloff et al. 1997). Furthermore, the pRAP15 plasmid has the Basta® selectable marker encoded by the *bar* gene which confers resistance to the herbicide bialphos, useful for tissue culture (Thompson et al. 1987; Rathore et al. 1993; Karimi et al. 2002, 2007). The *bar* gene is driven by the nopaline synthase promoter and terminated by the nopaline synthase terminator (Klink et al. 2021a).

### Genetic transformation of *Agrobacterium rhizogenes*

The pRAP15-*MAPK3-1* and -*MAPK3-2*-containing plasmids are genetically transformed into *Agrobacterium rhizogenes* strain 15,834 (15,834) using the freeze–thaw method (Hofgen and Willmitzer 1988; Pant et al. 2015). During the 15,834 genetic transformation procedure, 250 μl of bacteria previously snap frozen and stored at -80 °C in a 1:1 v/v cells in LB:30% sterile glycerol solution is thawed on ice. Plasmid DNA (0.1–1 μg) is added to 15,834 bacterial cells and gently mixed. The mixture of 15,834 cells and plasmid DNA is incubated on ice for 5 min. The contents are then subsequently transferred to liquid N_2_ for 5 min. The mixture is transferred to a 37 °C water bath for a period of 5 min. The reaction contents are then transferred to a culture tube containing 1 ml of LB medium with no antibiotics to allow for a recovery period for the bacteria as their TetR gene activity engages, placed in a shaking incubator at 28 °C, and incubated for 2 h. The 15,834 cells are then collected, centrifuged for 2 min at 5000 rpm. This step is followed by resuspension of the pelleted cells in 200 μl of LB medium, followed by the resuspended bacteria being spread on LB agar plates containing 5 μg/ml Tet for chemical selection at 28 °C (Pant et al. 2015). After 2 days the 15,834 colonies that underwent genetic transformation are picked to undergo a procedure that determines the presence of the e*GFP* gene, root inducing (Ri) plasmid, and the *MAPK3-1* or *MAPK3-2* gene by PCR using the appropriate primers (Hodges et al. 2004; Haseloff et al. 1997; Pant et al. 2016; McNeece et al. 2019) (Supplemental Table 1). The 15,834 colonies harboring the appropriate plasmids are then grown in 250 ml of LB medium containing 5 μg/ml Tet at 28 °C in a shaking incubator at 250 rpm for 14 h. Upon confirmation of adequate 15,834 growth at an OD_600_ of 0.6–0.8, the culture is used for *G. hirsutum* transformation after a centrifugation and resuspension in Murashige and Skoog (MS) media including vitamins (Duchefa, catalog number M0222), pH 5.7 at ambient room temperature (~ 18–21 °C) (Murashige and Skoog 1962). Please see the next section for details.

### Genetic transformation of *G. hirsutum*

*M. incognita*-susceptible *G. hirsutum* (Phytogen 565 WRF) seeds are planted in pre-wetted sterilized sand for germination. Seedlings are grown for 14 days at ambient greenhouse temperatures (~ 26–29 °C), then removed from the sand and washed in sterile, deionized water. The roots are excised with a sterile razor blade, producing root-less *G. hirsutum*. Genetic transformation of *G. hirsutum* is done as described by McNeece et al. (2017). An overnight culture of 15,834 containing the desired plasmid is grown in YEB Agrobacterium Growth Medium (Bioworld), supplemented with 5 μg/ml Tet at 28 °C (McNeece et al. 2017). The 15,834 cultures are pelleted during a 20 min spin at 4000 RPM in a Sorvall RC6 + centrifuge at 4 °C. The pellet is re-suspended in 25 mL of MS media including vitamins (Duchefa, catalog number M0222), pH 5.7 at ambient room temperature (~ 18–21 °C) (Murashige and Skoog 1962). Subsequently, 25 root-less *G. hirsutum* plants are grouped and placed in a 140 ml beaker containing 25 ml of 15,834 harboring the pRAP15-*MAPK3-1* or -*MAPK3-2* expression plasmids or pRAP15-*ccd*B control at ambient room temperature. The plants are placed under an ~ 15 psi (~ 103.42 kPa) vacuum for 20 min at ambient room temperature. After 20 min, the vacuum is slowly released over a period of 5 min at ambient room temperature. The root-less *G. hirsutum* plants are placed in 50 cell flats (T.O. Plastics) in coarse A-3 vermiculite (Palmetto Vermiculite) with one plant per cell at ambient room temperature. The 50-cell flats are placed in 24 L, 61.9 × 34.8 × 15.6 cm plastic containers (Sterlite) with the lid secured for 2 weeks in a culture room at ambient temperature (~ 20–24° C) while the plants are recovering under cool white fluorescent lights (Sylvania 21,781 FO32/841/ECO T8, 32 Watt, 4100 Kelvin, 2950 Lumens 48 inch tube bulbs, color rendering index [CRI] of 85) for 16 h day/8 h night at ambient room temperature. The recovered plants are placed in a greenhouse under ambient temperatures (~ 26–29 °C) for two weeks prior to selection of transgenic plants.

### Selection of transgenic *G. hirsutum*

The pRAP15 vector, containing the *eGFP* visual reporter gene, is used to accomplish the expression of a targeted CRG (Jefferson et al. 1987; Collier et al. 2005; Matsye et al. 2012; McNeece et al. 2017; Klink et al. 2021a). In control and experimental plants, the *eGFP* (driven by the *rol*D promoter), *ccd*B control gene (driven by the FMV-Sgt promoter), *MAPK3-1* (driven by the FMV-Sgt promoter), and *MAPK3-2* (driven by the FMV-Sgt promoter) genes that are engineered into the pRAP15 plasmid each have their own promoter and terminator sequences (Collier et al. 2005; Matsye et al. 2012; Pant et al. 2015, 2016; McNeece et al. 2017; Niraula et al. 2020a; Klink et al. 2021a). Due to the manner that 15,834 transfers the DNA cassettes located between the left and right borders of the destination vector into the root cell chromosomal DNA, the subsequent growth and development of the stably transformed genetically engineered cell into transgenic roots results in the production of a plant that is a genetic mosaic called a composite plant (Tepfer 1984; Collier et al. 2005). The composite, genetically mosaic, plant has a transgenic root system and a non-transgenic shoot.

### Experimental approach and replication

The *MAPK3-1* and *MAPK3-2* gene sequences are expressed in *G. hirsutum* using the pRAP15 plasmid vector to evaluate their effect(s) on *M. incognita* parasitism as compared to their respective pRAP15-*ccd*B control analyzed at each of 4 developmental stages. The analyses then determine the effect of *MAPK3-1* and *MAPK3-2* expression on *M. incognita* reproduction in comparison to their pRAP15-*ccd*B control through the calculation of the reproductive factor (RF), described in a later section (Oostenbrink 1966). The effects of *MAPK3-1* and *MAPK3-2* gene expression on *G. hirsutum* root mass as compared to their pRAP15-*ccd*B control is quantified using published methods that are described in a later section (Pant et al. 2015, 2016).

The experimental replicates (replicates) of the roots include *MAPK3-1*-E-replicate 1 (10 plants), *MAPK3-1*-E-replicate 2 (10 plants), and *MAPK3-1*-E-replicate 3 (10 plants) for a total of 30 *MAPK3-1*-E roots; *MAPK3-2*-E-replicate 1 (13 plants), *MAPK3-2*-E-replicate 2 (13 plants), and *MAPK3-2*-E-replicate 3 (14 plants) for a total of 40 *MAPK3-2*-E roots; and for the control, pRAP15-*ccd*B-E-replicate 1 (10 roots), pRAP15-*ccd*B-E-replicate 2 (10 roots), and pRAP15-*ccd*B-E-replicate 3 (10 roots) for a total of 30 total pRAP15-*ccd*B-E roots. Therefore the 3 biological replicates include a total of 30 *MAPK3-1*-E roots, a total of 40 *MAPK3-2*-E roots, and 30 pRAP15-*ccd*B-E roots.

### PCR

DNA primer sequences are provided (Supplemental Table 1). Isolated RNA (according to Invitrogen) is used to produce cDNA from transgenic *G. hirsutum* root RNA. Confirmation of *eGFP* expression is performed by PCR according to Niraula et al. (2020a). PCR using cDNA produced from mRNA isolated from the pRAP15-*ccd*B control and the *MAPK3-1* and *MAPK3-2*-expressing roots are used to demonstrate that *MAPK3-1* and *MAPK3-2* are expressed in the transgenic roots of *G. hirsutum*.

### Infection by *M. incognita*

The *M. incognita* (race 3) are confirmed by the North Carolina differential host test and increased on *Lycopersicum esculentum* (tomato) under ambient greenhouse conditions (Jenkins, 1964; Hussey and Barker 1973; Myers 1990; Tang et al. 1994; Diez et al. 2003). Eggs are extracted from roots by placing the root system in a 0.625% NaOCl solution and agitating the roots for 4 min using a rotary shaker at 120 rpm. Eggs are rinsed with tap water, collected on a 25-μm-pore sieve, then processed by sucrose centrifugation-flotation at 240 g for 1 min (Jenkins 1964). *M. incognita* eggs are placed in a modified Baermann funnel (Peraza-Padilla et al. 2013) on a slide warmer (Model 77) (Marshall Scientific, Brentwood, NH) and incubated at 31 °C for 5 to 7 days to obtain second stage juveniles (J2s) (Xiang et al. 2016). The J2s are collected on a 25-μm-pore sieve, transferred to 1.5 ml microcentrifuge tubes, centrifuged at 5000 g for 1 min, rinsed with sterile distilled water and centrifuged at 5000 g for 1 min. The J2 suspension is adjusted to 30 to 40 J2s per 10 μl of water (Xiang et al. 2016). *M. incognita* extraction is performed by gravity screening and centrifugal flotation (sucrose specific gravity = 1.13) (Jenkins 1964). *M. incognita* eggs and J2s are extracted from *L. esculentum* roots by a 4-min root immersion in 0.525% NaOCl (Hussey and Barker 1973). The hatched *M. incognita* J2s are maintained at 4 ± 1 °C in water until inoculation (Tang et al. 1993). Transgenic *G. hirsutum* plants are grown in 15 cm diameter clay pots. The pots are filled with 500 cm^3^ of the sterilized soil mixture that is 80% sand, 10% clay, and 10% silt. In these pots, a suspension of 2500 M*. incognita* J2s in 3 ml suspension are pipetted, divided into 2, 1.5 ml aliquots, into each of two 2.5 cm diameter × 2.5 cm deep depressions made into the soil. Once the 1.5 ml of inoculum is dispensed into each of the 2 depressions and absorbed into the soil, the holes are covered to prevent expulsion of the nematodes by subsequent watering. The plants are placed in the greenhouse, maintained at a temperature range of 25 °C–35 °C and given at least 12 h/day of ambient light supplemented with the cool white fluorescent lights, bringing the lighting to 16 h day/8 h night. The *M. incognita* life-stage development is described using a modified Christie’s method (Christie 1946; Christie and Wash 1946; Tang et al. 1994). The nematodes are extracted by combined gravity screening and sucrose centrifugation at 50 days post infection (dpi). The nematodes are enumerated on grated Petri dishes with an Olympus BH2 B071 microscope (Japan Model C35AD-4) at 40 X magnification (Aljaafri et al. 2017).

### Analysis of results

Root fresh weights are determined to allow the calculation of galls, egg masses, eggs, and J2s per gram of root tissue to standardize their presence in relation to the size (mass) of the root structure (see below) (Pant et al. 2016). The enumeration and statistical analyses of galls, egg masses, eggs, and J2s are done, analyzing them in two different ways. These two different analyses include enumerating the number of galls, egg masses, eggs, and J2s in relation to the whole root system (wr) which does not consider the effect the transgene has on root growth. A second analysis that does consider the effect that the transgene has on root growth is done by standardizing the number of galls and *M. incognita* per gram of root tissue (pg). In each analysis the results are considered statistically significant if *p* < 0.05, determined using Mann–Whitney-Wilcoxon Rank Sum Test (MWW) (Mann and Whitney 1947; Niraula et al. 2020a). The MWW Rank Sum Test is a nonparametric test of the null hypothesis not requiring the assumption of normal distributions (Mann and Whitney 1947).

### Calculation of the reproductive factor (RF)

The RF is calculated as RF = eggs + juveniles extracted at 60 days post inoculation/2500. The denominator (2500) represents the starting inoculum of 2500 J2s. Direct comparison of the effect that the expression of MAPK3-1 has to MAPK3-2 is done using the Dunn's multiple comparisons test (Dunn 1964).

## Results

### Analysis of MAPK3-1 and MAPK3-2 paralogs

The analysis begins by understanding the MAPK3-1 and MAPK3-2 paralogs better through an examination of their aa sequences. The analysis generates a 371 aa alignment having a 98.7% (366/371) identity and 98.9% (367/371) similarity with no gaps (Supplemental Fig. 1). At aa position 13 there is a P/A nonpolar to nonpolar R group difference, position 15 has a T/V nonpolar to nonpolar R group difference, position 36 has a A/T nonpolar to nonpolar R group difference; position 45 has a V/I nonpolar to nonpolar R group difference while position 142 has a S/C polar, but neutral to polar, but neutral R group difference. MAPK3-1 and MAPK3-2 have the TEY activation loop (A-loop) conserved domain (cd07858) at aa positions 197–199, consistent with plant MAPK3s.

**Fig. 1:**
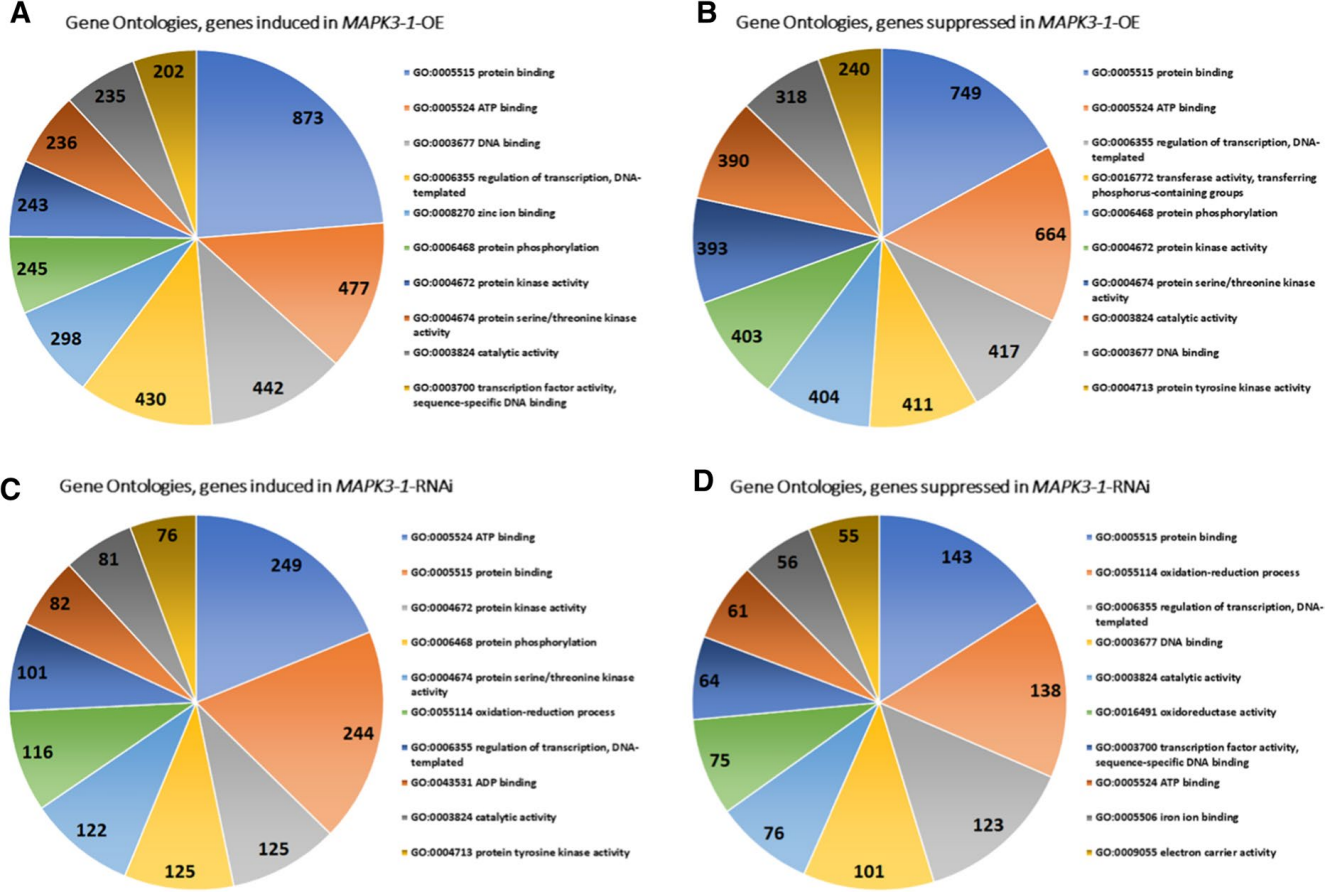
Gene Ontology analysis of the *MAPK3-1*-OE and *MAPK3-1*-RNAi induced and suppressed genes. **A** *MAPK3-1*-OE induced genes GO analysis **B** *MAPK3-1*-OE suppressed genes **C** *MAPK3-1*-RNAi induced genes GO analysis **D** *MAPK3-1*-RNAi suppressed genes GO analysis. Gene Ontologies, specifically molecular function, are retrieved from Phytozome, using the PhytoMine tool (https://phytozome.jgi.doe.gov/phytomine/begin.do) (Goodstein et al. 2012). Graphs are generated using Excel

Understanding the similarities and differences in MAPK3-1 and MAPK3-2 aa composition is important because the their proteins regulate the expression of proven defense genes occurring in common to them, and those regulated uniquely between them (McNeece et al. 2019; Klink et al. 2021a). RNA seq data for the *MAPK3-1* and *MAPK3-2* OE and RNAi roots and their respective pRAP15-*ccd*B (OE) and pRAP17-*ccd*B (RNAi) controls are analyzed (Alshehri et al. 2018). The numbers of induced and suppressed genes identified from the *MAPK3-1*-OE and *MAPK3-1*-RNAi roots, as well as the *MAPK3-2*-OE and *MAPK3-2*-RNAi roots, are presented (Table 1). The numbers of induced and suppressed genes expressed in common between the *MAPK3-1*-OE and *MAPK3-2*-OE, as well as the *MAPK3-1*-RNAi and *MAPK3-2*-RNAi roots, are presented as compared to their respective controls (Table 1). The top 10 induced or suppressed genes for the *MAPK3-1*-OE or *MAPK3-1*-RNAi roots as compared to their pRAP15-*ccd*B or pRAP17-*ccd*B control, respectfully, are presented (Table 2; Supplemental Tables 2, 3). A list limited to the top 10 induced or suppressed genes for the *MAPK3-2*-OE or *MAPK3-2*-RNAi roots as compared to their pRAP15-*ccd*B or pRAP17-*ccd*B control, respectfully, is presented (Table 2; Supplemental Table 4, 5). A list limited to the top 10 most highly induced or suppressed genes occurring in common between the *MAPK3-1*-OE and *MAPK3-2*-OE roots in comparison to the pRAP15-*ccd*B control is presented (Table 3). A list limited to the top 10 most highly induced or suppressed genes occurring in common between the *MAPK3-1-*RNAi and *MAPK3-2*-RNAi roots as compared to the pRAP17-*ccd*B control is presented (Table 3). A complete gene list of the induced or suppressed genes expressed in the *MAPK3-1*-OE and *MAPK3-2*-OE roots in comparison to the pRAP15-*ccd*B control, as well as the *MAPK3-1*-RNAi and *MAPK3-2*-RNAi roots to the pRAP17-*ccd*B control is presented (Supplemental Tables 6 and 7).

**Table 1:** Presented are gene counts for the *MAPK3-1*-OE and *MAPK3-1*-RNAi roots compared to their pRAP15-*ccd*B (overexpression) and pRAP17-*ccd*B (RNAi) control, as well as the *MAPK3-2*-OE and *MAPK3-2*-RNAi roots as compared to their pRAP15-*ccd*B and pRAP17-*ccd*B control, and genes in common between the two

Gene count	MAPK3-1-OE	MAPK3-2-OE	Common-OE	Common-OE-highly
Induced	4669	5129	1930	76
Suppressed	5611	5487	3314	115

Gene count	MAPK3-1-RNAi	MAPK3-2-RNAi	Common-RNAi	Common-RNAi-highly
Induced	1348	5668	812	14
Suppressed	1189	5085	696	1

**Table 2:** Presented are the top 10 most highly induced or most highly suppressed genes for *MAPK3-1*-OE, *MAPK3-1*-RNAi, *MAPK3-2*-OE, *MAPK3-2*-RNAi analyses as compared to their respective pRAP15-*ccd*B-OE or pRAP17-*ccd*B-RNAi controls

Analysis type	Gene name	M	Probability	Gene description
MAPK3-1-O-I	Glyma.01G118000	11.13887385	0.999965546	Thiamine pyrophosphate dependent pyruvate decarboxylase
	Glyma.03G221350	11.07438979	0.999800404	glycerol-3-phosphate acyltransferase 2
	Glyma.03G221350	11.07438979	0.999800404	AMP-dependent synthetase and ligase protein
	Glyma.03G220751	11.00644046	0.999784959	
	Glyma.14G176800	10.53199868	0.999610312	Protein of unknown function (DUF1637)
	Glyma.04G213900	9.925603452	1	alcohol dehydrogenase 1
	Glyma.16G037600	9.86286902	1	Protein of unknown function (DUF1637)
	Glyma.05G123700	9.679251852	0.9989343	polygalacturonase inhibiting protein 1
	Glyma.05G230300	9.625563981	0.998836878	
	Glyma.08G012900	9.569800709	0.998764405	nucleotide binding
MAPK3-1-O-S	Glyma.15G062800	− 11.85480717	1	CAP (Cysteine-rich secretory proteins, Antigen 5, Pathogenesis-related 1)
	Glyma.19G151200	− 11.47798233	0.999891885	Disease resistance-responsive (dirigent-like protein) protein
	Glyma.04G113400	− 10.98440838	0.999781395	FAD-binding Berberine protein
	Glyma.13G252400	− 10.97208198	0.999989307	CAP (Cysteine-rich secretory proteins, Antigen 5, Pathogenesis-related 1)
	Glyma.19G151100	− 10.92629967	0.999976239	Disease resistance-responsive (dirigent-like protein) family protein
	Glyma.13G251700	− 10.84098913	0.99972912	CAP (Cysteine-rich secretory proteins, Antigen 5, Pathogenesis-related 1)
	Glyma.U039500	− 10.74842301	0.999694666	Pseudouridine synthase protein
	Glyma.02G156100	− 10.61357703	0.999897826	Cytochrome p450, family 71, subfamily B, polypeptide 11
	Glyma.17G014400	− 9.893019833	0.999152905	
	Glyma.17G014100	− 9.850461739	0.999921587	
MAPK3-1-R-I	Glyma.19G069300	7.745442218	0.989701409	Protein kinase
	Glyma.10G098400	7.202649172	0.98195114	Protein kinase
	Glyma.03G054100	6.289063923	0.953146207	Disease resistance protein (TIR-NBS-LRR class)
	Glyma.18G254300	6.255116591	0.951419245	Leucine-rich repeat receptor-like protein kinase family protein
	Glyma.07G178200	6.148201388	0.945461459	Cupredoxin protein
	Glyma.14G015300	6.110726682	0.945461459	multidrug resistance-associated protein 3
	Glyma.18G250500	6.072252534	0.943150653	Leucine-rich repeat receptor-like protein kinase
	Glyma.02G028400	5.95026201	0.93525764	
	Glyma.12G054700	5.816995479	0.9254294	lipoxygenase 2
	Glyma.05G204800	5.782405297	0.979021388	osmotin 34
MAPK3-1-R-S	Glyma.14G200900	− 8.24082926	0.997733655	O-methyltransferase
	Glyma.17G011100	− 6.874809738	0.97459751	Stigma-specific Stig1 protein
	Glyma.15G145600	− 5.918901165	0.97171573	MLP-like protein 423
	Glyma.06G195000	− 4.929548409	0.984304067	expansin A15
	Glyma.13G112400	− 4.863343188	0.999120139	Integrase-type DNA-binding protein
	Glyma.03G173200	− 4.674975582	0.902971873	C2H2 and C2HC zinc fingers protein
	Glyma.19G175200	− 4.636501434	0.992576169	exocyst subunit exo70 family protein H4
	Glyma.10G262600	− 4.621426114	0.997790986	plant U-box 22
	Glyma.15G180000	− 4.471442188	0.998958675	Integrase-type DNA-binding superfamily protein
	Glyma.19G132500	− 4.427048068	0.951391164	basic helix-loop-helix (bHLH) DNA-binding protein
MAPK3-2-O-I	Glyma.18G033200	9.804556228	0.999903986	Bifunctional inhibitor/lipid-transfer protein/seed storage 2S albumin protein
	Glyma.16G148300	9.365338569	0.999130017	spermidine hydroxycinnamoyl transferase
	Glyma.03G058950	9.03276323	0.99873308	glycosyl hydrolase 9B7
	Glyma.09G129100	8.804556228	0.999674488	WRKY family transcription factor
	Glyma.10G177400	8.793575566	0.998289307	Protein of unknown function (DUF1442)
	Glyma.13G222100	8.408839339	1	senescence-related gene 1
	Glyma.19G199900	8.375364635	0.999432111	Aluminium activated malate transporter protein
	Glyma.05G036300	8.365338569	0.997186314	spermidine synthase 1
	Glyma.07G034900	8.277875728	0.99687017	lipoxygenase 1
	Glyma.07G092700	8.266560414	0.99683153	BR enhanced expression 1
MAPK3-2-O-S	Glyma.13G252400	− 12.18443627	0.999995316	CAP (Cysteine-rich secretory proteins, Antigen 5, Pathogenesis-related 1)
	Glyma.02G156100	− 11.51884455	0.9999356	cytochrome p450, family 71, subfamily B, polypeptide 11
	Glyma.15G156100	− 11.46107059	1	cytochrome P450, family 81, subfamily D, polypeptide 3
	Glyma.15G062800	− 11.06282253	1	CAP (Cysteine-rich secretory proteins, Antigen 5, Pathogenesis-related 1)
	Glyma.U033205	− 10.92877516	0.999868859	disease resistance protein (TIR-NBS-LRR class), putative
	Glyma.13G251700	− 10.84179186	0.99985832	CAP (Cysteine-rich secretory proteins, Antigen 5, Pathogenesis-related 1)
	Glyma.18G239100	− 10.80672821	0.999850124	S-adenosyl-L-methionine-dependent methyltransferase
	Glyma.13G162700	− 10.79414648	0.999847782	RING/U-box superfamily protein
	Glyma.10G184600	− 10.58381569	0.999995316	Serine protease inhibitor, potato inhibitor I-type protein
	Glyma.16G170000	− 10.31603372	1	
MAPK3-2-R-I	Glyma.02G240600	13.85812251	1	glutathione S-transferase TAU 19
	Glyma.09G201500	13.68881465	1	Concanavalin A-like lectin protein kinase
	Glyma.11G095900	13.62842779	1	Bifunctional inhibitor/lipid-transfer protein/seed storage 2S albumin protein
	Glyma.08G274700	13.55386329	1	Concanavalin A-like lectin protein kinase
	Glyma.09G201600	13.35615704	1	Concanavalin A-like lectin protein kinase
	Glyma.14G123500	13.30236808	1	phosphate transporter 1;1
	Glyma.09G201400	13.27673576	1	Concanavalin A-like lectin protein kinase
	Glyma.14G210100	13.26262798	1	glutathione S-transferase TAU 19
	Glyma.13G291100	13.07694796	0.999998826	Protein of unknown function, DUF538
	Glyma.12G210200	12.89925823	0.999997653	Protein of unknown function, DUF538
MAPK3-2-R-S	Glyma.03G176300	− 9.17880158	0.997912021	Glutathione S-transferase
	Glyma.04G113400	− 9.031525318	0.997551701	FAD-binding Berberine protein
	Glyma.20G036100	− 8.873687809	0.999893195	ribonuclease 1
	Glyma.19G176600	− 8.665097763	0.996298209	Protein phosphatase 2C
	Glyma.01G021000	− 8.57546055	0.995956668	elicitor-activated gene 3–2
	Glyma.10G016600	− 8.366873928	0.99490857	Pollen Ole e 1 allergen and extensin
	Glyma.15G062800	− 7.970952843	0.999913148	CAP (Cysteine-rich secretory proteins, Antigen 5, Pathogenesis-related 1)
	Glyma.15G103000	− 7.85991394	0.991189174	Family of unknown function (DUF716)
	Glyma.03G215900	− 7.833172649	0.997895589	Plant invertase/pectin methylesterase inhibitor superfamily
	Glyma.19G144800	− 7.813620287	0.990732612	geranylgeranyl pyrophosphate synthase 1

Analyzed samples: *MAPK3-1*-O-I, *MAPK3-1* overexpression, induced genes; *MAPK3-1*-O-S, *MAPK3-1* overexpression, suppressed genes; *MAPK3-1*-R-I, *MAPK3-1*-RNAi, induced genes; *MAPK3-1*-R-S, *MAPK3-1*-RNAi, suppressed genes; *MAPK3-2*-O-I, *MAPK3-2* overexpression, induced genes; *MAPK3-2*-O-S, *MAPK3-2* overexpression, suppressed genes; *MAPK3-2*-R-I, *MAPK3-2*-RNAi, induced genes; *MAPK3-2*-RNAi-S, *MAPK3-2*-RNAi, suppressed genes. M, relative fold change in transcript abundance

**Table 3:** Presented are the top 10 most highly induced or suppressed genes for *MAPK3-1*-OE and *MAPK3-2*-OE expressed in common in comparison to the pRAP15-*ccd*B control and also genes in common between *MAPK3-1*-RNAi and *MAPK3-2*-RNAi analyses as compared to the pRAP17-*ccd*B control

Analysis type	Gene name	M (MAPK3− 1)	Probability	M (MAPK3− 2)	Probability	Gene description
MAPK3-1-MAPK3-2-O-I	Glyma.03G221350	11.07438979	0.999800404	7.277875728	0.989800244	glycerol-3-phosphate acyltransferase 2
	Glyma.05G123700	9.679251852	0.9989343	5.67019315	0.928527938	polygalacturonase inhibiting protein 1
	Glyma.05G123900	9.384390768	0.999697042	5.711044343	0.986609526	polygalacturonase inhibiting protein 1
	Glyma.20G098300	8.981408771	1	5.093513979	0.99970259	Inorganic H pyrophosphatase family protein
	Glyma.15G052600	8.727888223	0.99999406	6.176707915	0.999894619	Peroxidase superfamily protein
	Glyma.03G079150	8.711819714	0.996805275	7.300243541	0.990001639	n/a
	Glyma.08G179800	7.82332803	0.991714388	5.525803241	0.918819962	Peroxidase superfamily protein
	Glyma.18G263200	7.799282556	0.991449448	7.365338569	0.99059763	multidrug resistance-associated protein 3
	Glyma.16G038000	7.396317889	0.986933587	6.599803823	0.97683598	dehydrin family protein
	Glyma.16G037800	7.395267357	0.996973981	6.645156407	0.99571917	Plant protein of unknown function (DUF639)
MAPK3-1-MAPK3-2-O-S	Glyma.15G062800	− 11.8548072	1	− 11.06282253	1	CAP (Cysteine-rich secretory protein, Antigen 5, Pathogenesis-related 1)
	Glyma.19G151200	− 11.4779823	0.999891885	− 6.615946832	0.999891885	Disease resistance-responsive (dirigent-like protein) family protein
	Glyma.04G113400	− 10.9844084	0.999781395	− 5.247903758	0.999781395	FAD-binding Berberine family protein
	Glyma.13G252400	− 10.972082	0.999989307	− 12.18443627	0.999989307	CAP (Cysteine-rich secretory protein, Antigen 5, Pathogenesis-related 1)
	Glyma.19G151100	− 10.9262997	0.999976239	− 8.231763364	0.999976239	Disease resistance-responsive (dirigent-like protein) family protein
	Glyma.13G251700	− 10.8409891	0.99972912	− 10.84179186	0.99972912	CAP (Cysteine-rich secretory protein, Antigen 5, Pathogenesis-related 1)
	Glyma.02G156100	− 10.613577	0.999897826	− 11.51884455	0.999897826	cytochrome p450, family 71, subfamily B, polypeptide 11
	Glyma.10G176700	− 9.31126913	0.999983367	− 10.16105334	0.999983367	O-methyltransferase family protein
	Glyma.03G032400	− 9.16519087	0.999961982	− 8.529548258	0.999961982	SPX domain gene 3
	Glyma.07G262400	− 8.99248074	0.999155281	− 6.297944441	0.999155281	F-box family protein with a domain of unknown function (DUF295)
MAPK3-1-MAPK3-2-R-I	Glyma.10G098400	7.202649172	0.98195114	7.900217783	0.991523673	Protein kinase superfamily protein
	Glyma.18G254300	6.255116591	0.951419245	5.930591432	0.938551912	Leucine-rich repeat receptor-like protein kinase
	Glyma.14G015300	6.110726682	0.945461459	8.505500268	0.995629211	multidrug resistance-associated protein 3
	Glyma.12G054700	5.816995479	0.9254294	7.073549386	0.980062675	lipoxygenase 2
	Glyma.05G204800	5.782405297	0.979021388	6.498514096	0.990454449	osmotin 34
	Glyma.13G065451	5.720780164	0.917502457	6.515553933	0.965221474	n/a
	Glyma.20G229700	5.681476388	0.976352553	7.810458102	0.997720711	n/a
	Glyma.18G067200	5.506655358	0.902978893	5.433091772	0.902032816	alpha-glucan phosphorylase 2
	Glyma.16G151500	5.479842527	0.970934151	7.980383103	0.998109199	NAC domain containing protein 47
	Glyma.13G267600	5.475025511	0.997618992	7.490198828	0.999863853	WRKY DNA-binding protein 62
MAPK3-1-MAPK3-2-R-S	Glyma.15G145600	− 5.91890116	0.97171573	− 6.781911428	0.973329265	MLP-like protein 423

Analyzed samples: *MAPK3-1*-*MAPK3-2*-O-I, *MAPK3-1* and *MAPK3-2* overexpression, induced genes; *MAPK3-1*-*MAPK3-2*-O-S, *MAPK3-1* and *MAPK3-2* overexpression, suppressed genes; *MAPK3-1*-*MAPK3-2*-R-I, *MAPK3-1* and *MAPK3-2* RNAi, induced genes; *MAPK3-1*-*MAPK3-2*-RNAi-S, *MAPK3-1* and *MAPK3-2* RNAi, suppressed genes

**Fig. 2:**
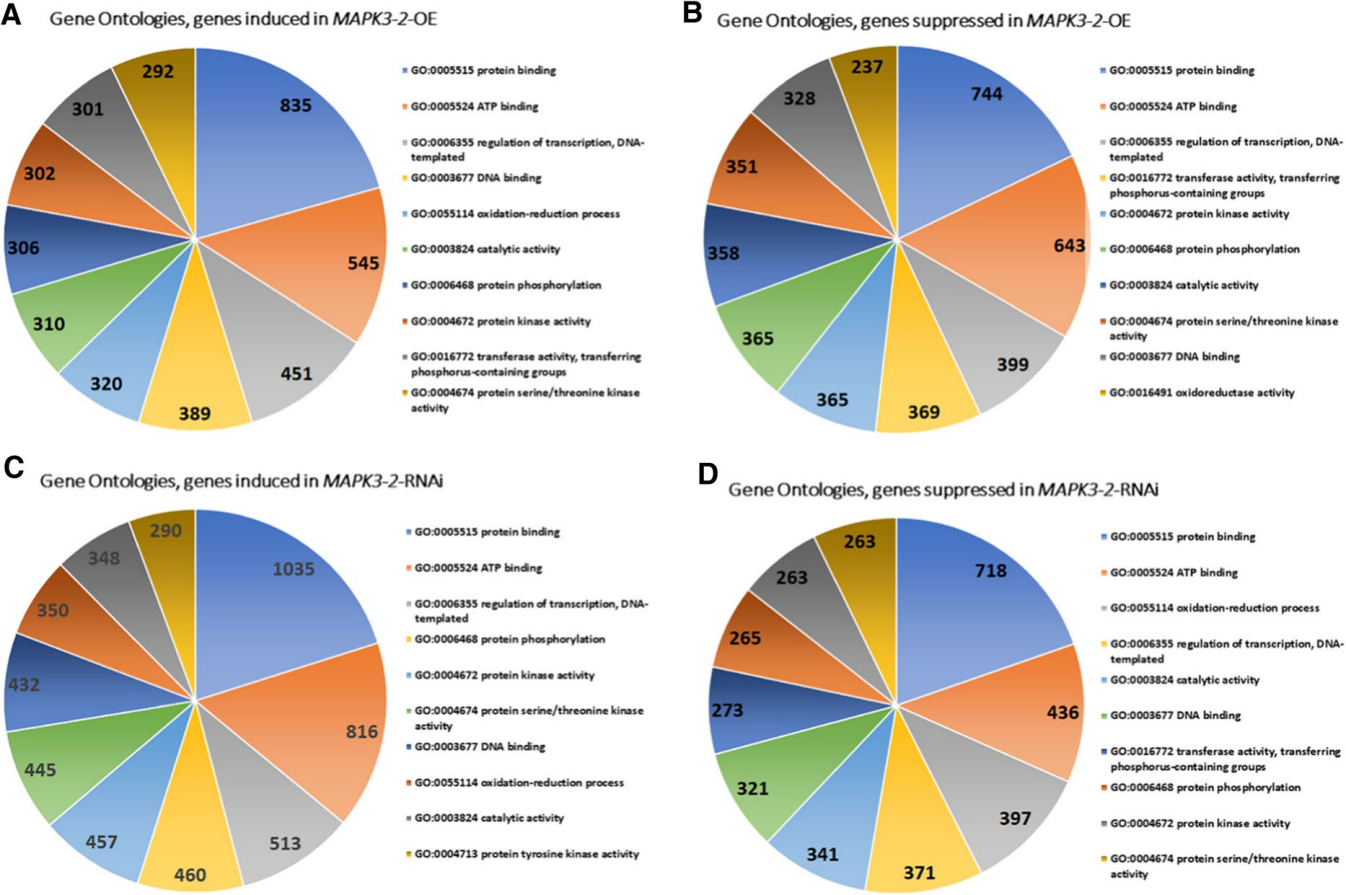
Gene Ontology analysis of the *MAPK3-2*-OE and *MAPK3-2*-RNAi induced and suppressed genes. **A** *MAPK3-2*-OE induced genes GO analysis **B** *MAPK3-2*-OE suppressed genes **C** *MAPK3-2*-RNAi induced genes GO analysis **D** *MAPK3-2*-RNAi suppressed genes GO analysis. Gene Ontologies, specifically molecular function, are retrieved from Phytozome, using the PhytoMine tool (https://phytozome.jgi.doe.gov/phytomine/begin.do) (Goodstein et al. 2012). Graphs are generated using Excel

**Fig. 3:**
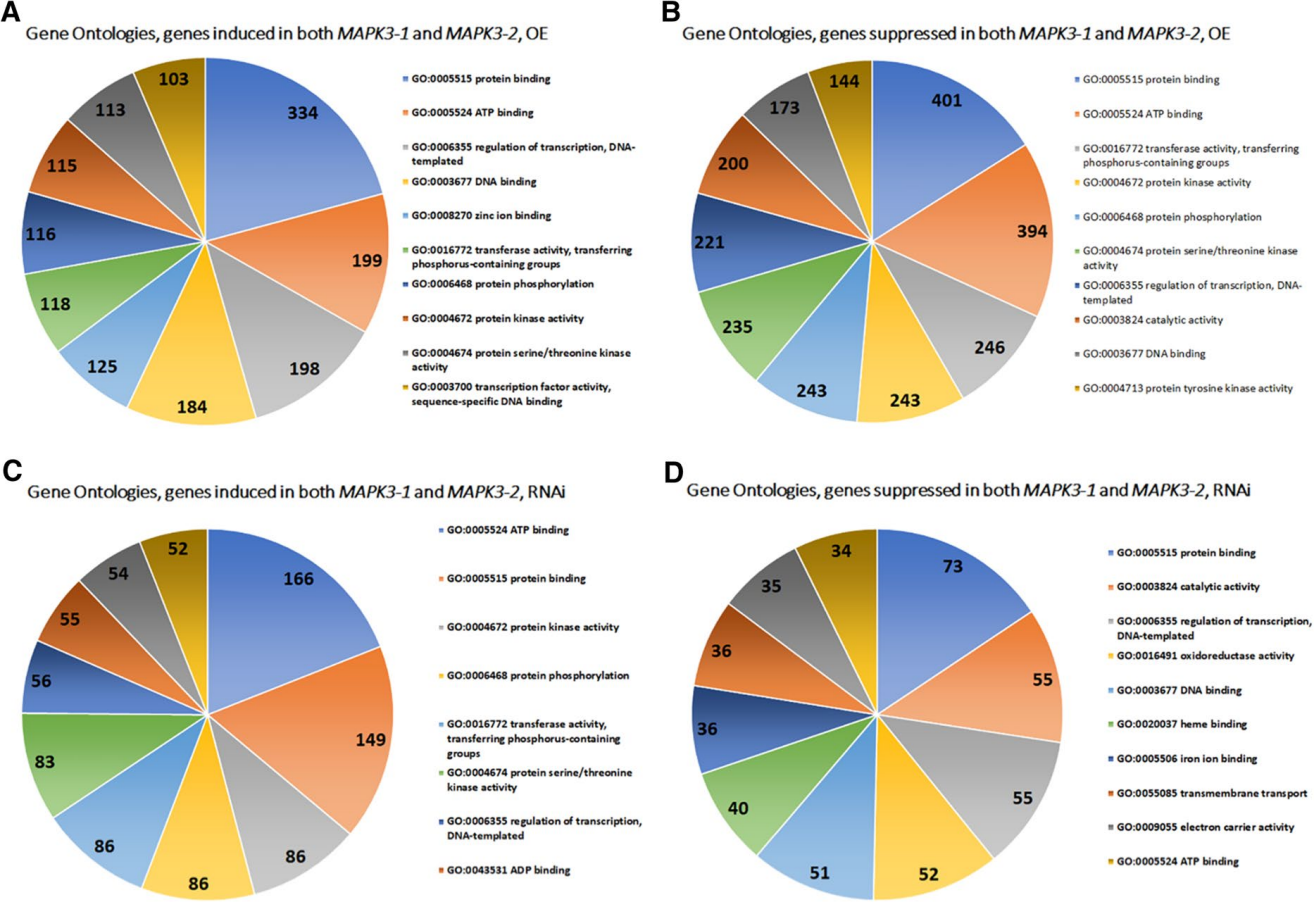
Gene Ontology analysis of the *MAPK3-1*-OE and *MAPK3-2*-OE genes expressed in common, and *MAPK3-1*-RNAi and *MAPK3-2*-RNAi genes expressed in common, induced and suppressed genes. **A** *MAPK3-1*-OE and *MAPK3-2*-OE induced genes GO analysis-**B** *MAPK3-1*-OE and *MAPK3-2*-OE suppressed genes **C** *MAPK3-1*-RNAi and *MAPK3-2*-RNAi induced genes GO analysis **D** *MAPK3-1*-RNAi and *MAPK3-2*-RNAi suppressed genes GO analysis. Gene Ontologies, specifically molecular function, are retrieved from Phytozome, using the PhytoMine tool (https://phytozome.jgi.doe.gov/phytomine/begin.do) (Goodstein et al. 2012). Graphs are generated using Excel

GO analyses presented here using RNA seq data obtained from *MAPK3-1*-OE or *MAPK3-1*-RNAi roots as compared to their pRAP15-*ccd*B or pRAP17-*ccd*B controls, respectively (Fig. 1; Supplemental Tables 2, 3). The same comparisons are made for *MAPK3-2*-OE or *MAPK3-2*-RNAi roots as compared to the pRAP15-*ccd*B or pRAP17-*ccd*B controls, respectively (Fig. 2; Supplemental Tables 4 and 5). GO analyses of induced or suppressed genes existing in common between the *MAPK3-1*-OE and *MAPK3-2*-OE lists in comparison to their pRAP15-*ccd*B control lists, and *MAPK3-1*-RNAi and *MAPK3-2*-RNAi lists in comparison to their pRAP17-*ccd*B control lists are provided (Fig. 3; Supplemental Tables 6 and 7).

**Table 4:** *G. hirsutum MAPK3* genes as identified by comparison to the *G. max MAPK3-1* and *G. max MAPK3-2* protein sequences

*G. max* MAPK3-1	Percent identity	*G. max* MAPK3-2	Percent identity
Gohir.D03G132800.1.p	85	Gohir.D03G132800.1.p	85
Gohir.A03G035400.1.p	85	Gohir.A03G035400.1.p	85
Gohir.D05G100500.1.p	85	Gohir.D05G100500.1.p	85
Gohir.A03G088300.1.p	82	Gohir.A03G088300.1.p	83
Gohir.D02G108500.1.p	79	Gohir.D02G108500.1.p	79
Gohir.A02G009100.1.p	79	Gohir.A02G009100.1.p	79

Comparison of the MAPK3-1 and MAPK3-2 protein sequences to the *G. hirsutum* proteome leads to the identification of the same 6 paralogs, including 3 from its A genome (Gohir.A03G035400.1.p, Gohir.A02G009100.1.p, Gohir.A03G088300.1.p) and 3 from its D genome (Gohir.D03G132800.1.p, Gohir.D05G100500.1.p, Gohir.D02G108500.1.p) with aa identities to the MAPK3-1 and MAPK3-2 proteins of 79–85% (Table 3). The chromosomal location of these *G. hirsutum* genes, not in tandemly repeated arrangement(s), indicate they likely are not the product of localized duplication which is important for certain genes functioning in the defense process that *G. max* has toward *H. glycines* (Cook et al. 2012). Like *G. max*, *G. hirsutum* MAPK3 paralogs each have the TEY A-loop (Supplemental Fig. 2).

The high degree of sequence identity occurring between the *G. max* and *G. hirsutum* MAPK protein sequences lead to the hypothesis that the heterologous expression of the *G. max MAPK3s* in *G. hirsutum* will result in suppressing *M. incognita* parasitism. Furthermore, any significant differences in outcome occurring between the heterologous expression of *MAPK3-1* and *MAPK3-2* is limited to a relatively small number of nucleotides. This is because the expression of each gene is driven by the same FMV-Sgt promoter.

### *MAPK3-1* and *MAPK3-2* can be expressed in *G. hirsutum* roots

**Fig. 4:**
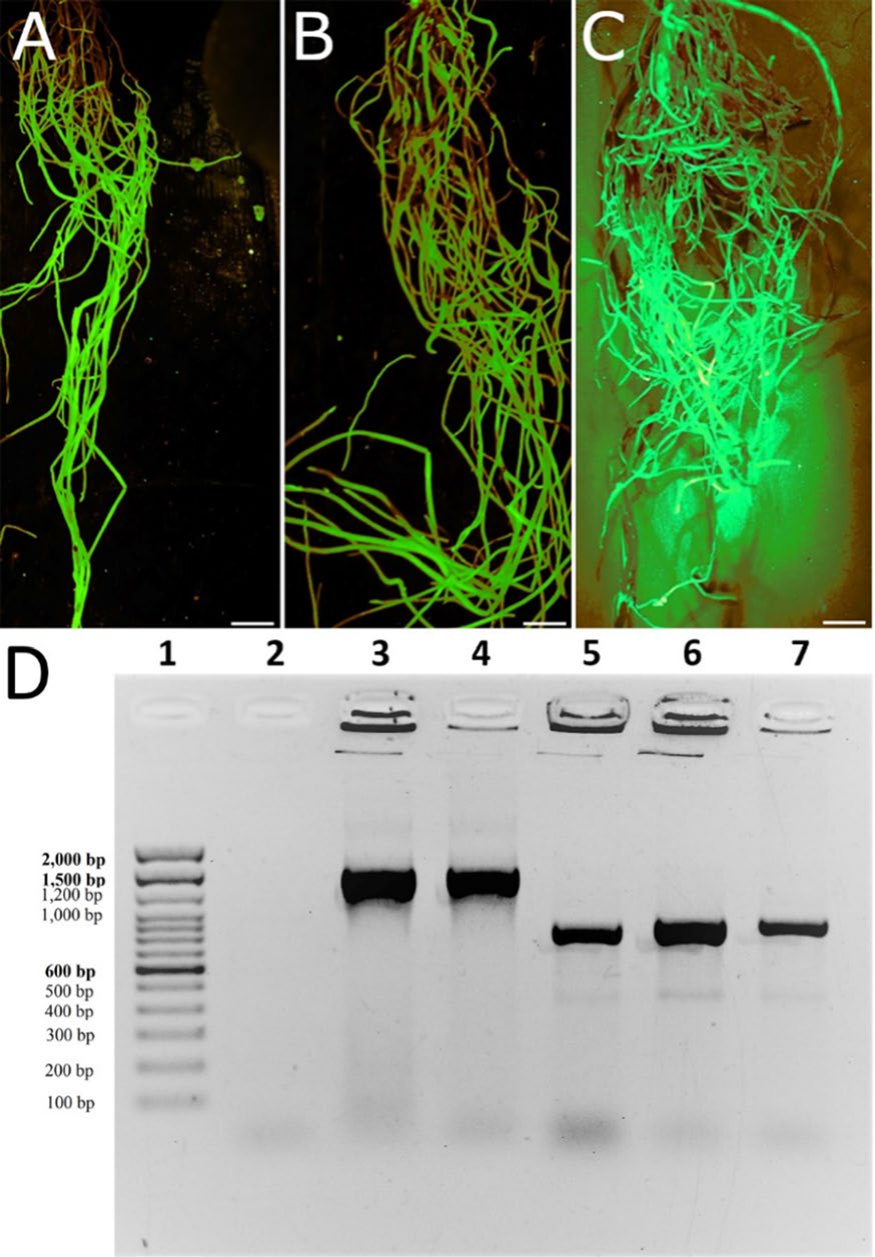
Generation of transgenic roots. **A** a representative pRAP15-*ccd*B control root revealed by the eGFP reporter. Bar = 1 cm. **B** a representative *G. max MAPK3-1*-expressing engineered root revealed by the eGFP reporter. Bar = 1 cm. **C** a representative *G. max MAPK3-2*-expressing engineered root revealed by the eGFP reporter. Bar = 1 cm. **D** representative PCR has been used to demonstrate the presence of the *MAPK3-1* and *MAPK3-2* transcript only in the transgenic *MAPK3-1* and *MAPK3-2* expressing lines in comparison to the control. **1** DNA ladder with base pairs indicated; **2** no template control; **3** *MAPK3-1*-expressing transgenic *G. hirsutum* root; **4** *MAPK3-2*-expressing transgenic *G. hirsutum*; **5** *eGFP* from CDNA made from an eGPF fluorescing *MAPK3-1*-expressing transgenic *G. hirsutum* root; **6** *MAPK3-2*-expressing transgenic *G. hirsutum* root; **7** transgenic *G. hirsutum* root engineered only with the pRAP15-*ccd*B *eGFP* expressing root. The *MAPK3-1* transcript is 1486 base pairs (bp). The *MAPK3-2* transcript cDNA is 1488 bp. The *eGFP* transcript cDNA is 864 bp

*G. hirsutum* is employed for a hairy root transgenesis procedure with the objective of expressing (E) the *MAPK3-1*-E or *MAPK3-2*-E cassettes in their roots using the pRAP15 expression plasmid (Supplemental Fig. 3) (Matsye et al. 2012). If needed, an RNA interference (RNAi) plasmid (pRAP17) was available (Klink et al. 2009). Steps in the hairy root procedure are presented (Supplemental Fig. 5) (Pant et al. 2015). Prior analyses show in rare cases that certain genes may not be able to undergo engineered expression, leading to the failure to obtain transgenic roots butthe results presented here show that *G. hirsutum* is expressing the *MAPK3-1*-E or *MAPK3-2*-E cassettes (Fig. 4) (Austin et al. 2019). These genetically mosaic, composite plants have the entire shoot being non-transgenic while the entire root system is transgenic (Tepfer 1984; Haas et al. 1995; Collier et al. 2005). Consequently, each individual transgenic root system functions as an independent transformant line (Tepfer 1984; Matsye et al. 2012; Matthews et al. 2013; Pant et al. 2014; McNeece et al. 2017). For reference, the numbers of studied transgenic roots are presented in the Materials and Methods section, Subsection *G. hirsutum* genetic transformation and the respective figure captions.

### *MAPK3-1* and *MAPK3-2* expression in *G. hirsutum* suppresses *M. incognita* gall production

**Fig. 5:**
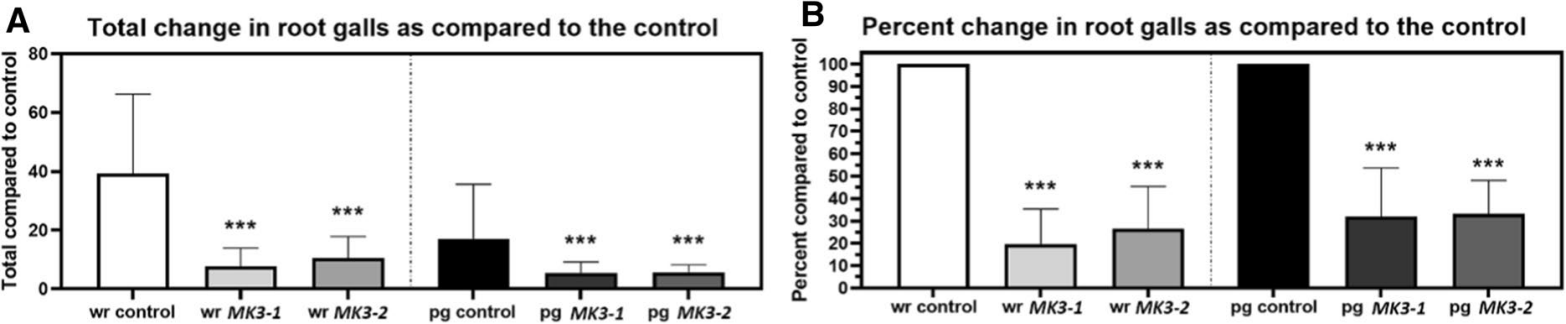
*M. incognita* gall analyses in whole roots (wr) and per gram (pg) of root tissue show *G. hirsutum* roots genetically engineered to express *MAPK* (*MK*) *MK3-1* and *MK3-2* affects their parasitism. **A** Total change in galls as compared to the control. **B** Transformed data From **A** showing percent change in galls as compared to the control. *, **, and *** denote statistical significance at the 0.05, 0.01, 0.001 probability level, respectively. Significance determined using Mann–Whitney-Wilcoxon Rank Sum Test (Mann and Whitney 1947). The number of experimental replicates, spanning the 3 biological replicates, include 30 *MAPK3-1*-E roots, 40 *MAPK3-1*-E roots, and 30 total pRAP15-*ccd*B-E roots. Please refer to Materials and Methods section, subsection: *G. hirsutum* genetic transformation, for details

*M. incognita* derives its nourishment from giant cells that are contained within an enlarged root structure called a gall, permitting an estimation of successful parasitism in many species of plants including *G. hirsutum*. Experiments presented here show that gall production normally induced by *M. incognita* is suppressed in *G. hirsutum* roots expressing the *MAPK3-1*-E or *MAPK3-2*-E cassettes as compared to the pRAP15-*ccd*B control in analyses of the whole root (wr) system (Fig. 5). To standardize the results, accounting for any developmental effects exerted on the *G. hirsutum* roots by the expression of the *MAPK3-1* or *MAPK3-2* transgenes, the number of *M. incognita*-induced galls occurring per gram (pg) of root tissue are calculated as compared to the pRAP15-*ccd*B control, also showing a reduction in gall number; *p* < 0.05, MWW Rank Sum Test. (Fig. 5). The effect that the expression of the *MAPK3-1*-E as compared to *MAPK3-2*-E cassettes has on gall production is not significantly different from each other in wr (*p* = 0.3492) and pg (*p* > 0.9999) analyses, Dunn’s multiple comparisons test.

### *MAPK3-1* and *MAPK3-2* expression in *G. hirsutum* suppresses *M. incognita* egg mass production

**Fig. 6:**
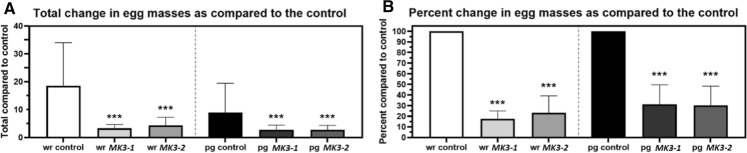
*M. incognita* egg mass analyses in whole roots (wr) and per gram (pg) of root tissue show *G. hirsutum* roots genetically engineered to express *MAPK* (*MK*) *MK3-1* and *MK3-2* affects their parasitism. **A** Total change in egg masses as compared to the control. **B** Transformed data From **A** showing percent change in egg masses as compared to the control. *, **, and *** denote statistical significance at the 0.05, 0.01, 0.001 probability level, respectively. Significance determined using Mann–Whitney-Wilcoxon Rank Sum Test (Mann and Whitney 1947). The number of experimental replicates, spanning the 3 biological replicates, include 30 *MAPK3-1*-E roots, 40 *MAPK3-1*-E roots, and 30 total pRAP15-*ccd*B-E roots. Please refer to Materials and Methods section, subsection: *G. hirsutum* genetic transformation, for details

Analyses are performed here to assess sexual maturity by determining the number of egg masses that are made by female *M. incognita*. The analyses show that the production of egg masses is also suppressed in *G. hirsutum* roots engineered with the *MAPK3-1*-E or *MAPK3-2*-E cassettes in wr and pg analyses as compared to the pRAP15-*ccd*B control; *p* < 0.05, MWW Rank Sum Test (Fig. 6). The effect that the expression of the *MAPK3-1* as compared to *MAPK3-2* transgenes has on egg mass production is not significantly different from each other in wr (*p* = 0.5228) and pg (*p* > 0.9999) analyses, Dunn’s multiple comparisons test.

### *MAPK3-1* and *MAPK3-2* expression in *G. hirsutum* does not negatively affect *M. incognita* egg production

**Fig. 7:**
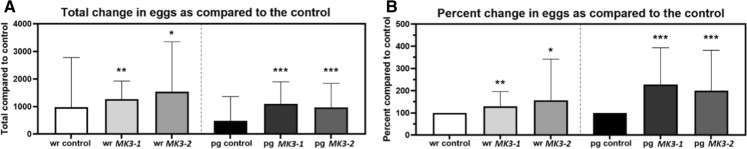
*M. incognita* egg analyses in whole roots (wr) and per gram (pg) of root tissue show *G. hirsutum* roots genetically engineered to express *MAPK* (*MK*) *MK3-1* and *MK3-2* affects their parasitism. **A**. Total change in eggs as compared to the control. **B**. Transformed data From **A** showing percent change in eggs as compared to the control. *, **, and *** denote statistical significance at the 0.05, 0.01, 0.001 probability level, respectively. Significance determined using Mann–Whitney-Wilcoxon Rank Sum Test (Mann and Whitney 1947). The number of experimental replicates, spanning the 3 biological replicates, include 30 *MAPK3-1*-E roots, 40 *MAPK3-1*-E roots, and 30 total pRAP15-*ccd*B-E roots. Please refer to Materials and Methods section, subsection: *G. hirsutum* genetic transformation, for details

In contrast to the results obtained for the number of galls and egg masses, the analyses reveal an increase in the number of *M. incognita* eggs in each of the roots genetically transformed with the *MAPK3-1*-E or *MAPK3-2*-E cassettes for the wr and pg analysis as compared to the pRAP15-*ccd*B control; *p* < 0.05, MWW Rank Sum Test (Fig. 7). The statistically significant results are those averaged from running the experiment in triplicate at different times, pointing to the validity of the outcome. The effect that the expression of the *MAPK3-1* as compared to *MAPK3-2* transgenes has on egg production is not significantly different from each other in wr (*p* > 0.9999) and pg (*p* > 0.9999) analyses, Dunn’s multiple comparisons test.

### *MAPK3-1* and *MAPK3-2* expression in *G. hirsutum* interferes with *M. incognita* J2 production

**Fig. 8:**
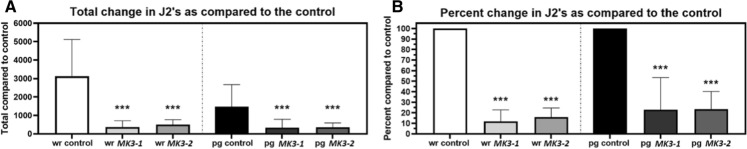
*M. incognita* J2 analyses in whole roots (wr) and per gram (pg) of root tissue show *G. hirsutum* roots genetically engineered to express *MAPK* (*MK*) *MK3-1* and *MK3-2* affects their parasitism. **A**. Total change in J2s as compared to the control. **B**. Transformed data From **A** showing percent change in J2s as compared to the control. *, **, and *** denote statistical significance at the 0.05, 0.01, 0.001 probability level, respectively. Significance determined using Mann–Whitney-Wilcoxon Rank Sum Test (Mann and Whitney 1947). The number of experimental replicates, spanning the 3 biological replicates, include 30 *MAPK3-1*-E roots, 40 *MAPK3-1*-E roots, and 30 total pRAP15-*ccd*B-E roots. Please refer to Materials and Methods section, subsection: *G. hirsutum* genetic transformation, for details

The number of *M. incognita* J2s extracted from the transgenic *MAPK3-1*-E and *MAPK3-2*-E roots are enumerated in as compared to the pRAP15-*ccd*B transgenic root control. The enumeration of J2s from *G. hirsutum* roots expressing the *MAPK3-1*-E or *MAPK3-2*-E cassettes reveals a significant decrease in the number of J2s in comparison to the number in pRAP15-*ccd*B control roots in wr and pg analyses; *p* < 0.05, MWW Rank Sum Test (Fig. 8). The effect that the expression of the *MAPK3-1* as compared to *MAPK3-2* transgene has on J2 production is not significantly different from each other in wr (*p* = 0.2104) and pg (*p* = 0.89) analyses, Dunn's multiple comparisons test.

### *MAPK3-1* and *MAPK3-2* expression in *G. hirsutum* decreases the *M. incognita* reproductive factor

An added analysis is performed to determine whether reproduction of *M. incognita* is detrimentally affected by the genetic engineering of the *MAPK3-1* or *MAPK3-2* transgenes into *G. hirsutum* as compared to the controls, respectively. The analysis is accomplished by calculating the RF where RF = eggs + juveniles extracted at 60 days post inoculation/2500 (J2 inoculum) (please refer to the Materials section for details). The analysis identifies an RF of 0.65 for *G. hirsutum* roots engineered with the *MAPK3-1* transgene and an RF of 0.81 for roots engineered with the *MAPK3-2* transgene. In contrast, the pRAP15-*ccd*B control RF is 1.6412. An RF > 1.0 indicates reproduction is occurring. The RF is calculated using the total number of eggs and J2s extracted from the soil and therefore an RF per gram of root analysis is not performed. The results show the increase in egg number is balanced off by a sharp decrease in the number of J2s.

### *MAPK3-1* and *MAPK3-2* expression in *G. hirsutum* affects its root growth

**Fig. 9:**
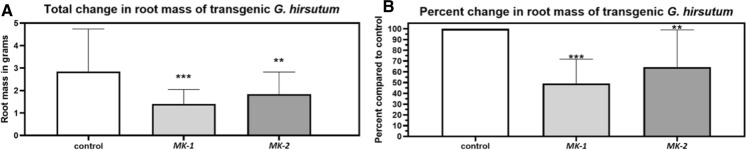
Root growth in relation to *MAPK3* (*MK3*) expression. Root growth is calculated as a percent with *MK3-1* and *MK3-2* average fresh weight divided by the pRAP15-*ccd*B control average fresh weight multiplied by 100. In each analysis the results are considered statistically significant if p < 0.05, determined using Mann–Whitney-Wilcoxon Rank Sum Test (Mann and Whitney 1947). **A**. Total change in root mass. **B**. Transformed data From A showing percent change in J2s as compared to the control. *, **, and *** denote statistical significance at the 0.05, 0.01, 0.001 probability level, respectively. The number of experimental replicates, spanning the 3 biological replicates, include 30 *MAPK3-1*-E roots, 40 *MAPK3-1*-E roots, and 30 total pRAP15-*ccd*B-E roots. please refer to Materials and Methods section, subsection: *G. hirsutum* genetic transformation, for details

Analyses of the mass of *G. hirsutum* roots expressing the *MAPK3-1*-E or *MAPK3-2*-E cassettes as compared to the pRAP15-*ccd*B control reveal a significant decrease in root mass; *p* < 0.05, MWW Rank Sum Test (Fig. 9). This result explains the differences occurring for *M. incognita*-induced galls, egg masses, eggs, and J2s that are observed between the wr and pg *MAPK3-1*-E and *MAPK3-2*-E analyses in comparison to their pRAP15-*ccd*B controls, respectively. However, the effect that the expression of the *MAPK3-1*-E as compared to *MAPK3-2*-E cassette has on root development, in relation to their pRAP15-*ccd*B control, is not significantly different from each other (*p* = 0.1983), Dunn's multiple comparisons test.

### MAPK3 homologs present in agriculturally important crops

The ability to heterologously express the *MAPK3-1* and *MAPK3-2* paralogs in *G. hirsutum*, leading to a defense response to *M. incognita*, indicates that the genes may function broadly in other plant species. Analyses of crops that are economically important worldwide and affected by climate change would aid from such transgenic studies like those done for *G. max* (Tilman et al. 2011; Liu et al. 2011; Neupane et al. 2013; Mohanta et al. 2015; Burkhead and Klink 2018; McNeece et al. 2019; Ray et al. 2013, 2019). MAPK homologs, including MAPK3, are identified in crops that are important world-wide to agriculture as well as to the U.S. (Supplemental Tables 8–20). The crops include *G. hirsutum, M. esculenta*, *Z. mays*, *O. sativa*, *T. aestivum*, *H. vulgare*, S*. bicolor*, *B. rapa*, *S. tuberosum*, *S. lycopersicum*, *E. guineensis, S. officinalis*, and *B. vulgaris*.

## Discussion

### MAPK3 is an important defense node

The MAPK3-1 and MAPK3-2 are shown to be 98.7% identical to each other. When BLASTing the *G. hirsutum* genome with the MAPK3-1 and MAPK3-2 protein sequences, the same 6 *G. hirsutum* paralogs with 3 from its A genome and 3 from its D genome having 79–85% identify are identified. The high level of primary aa sequence conservation, along with the presence of the TEY A-loops lead to the hypothesis that their expression would likely produce a defense response to *M. incognita* parasitism in *G. hirsutum*.

The RNA seq analyses identify defense genes that are induced or suppressed in expression. Recent experiments have examined a very narrow group of 309 *G. max* genes that are induced in their expression in each of 9 defense *MAPK*-OE root systems, including the overexpressed *MAPK2*, *MAPK3-1*, *MAPK3-2*, *MAPK4-1*, *MAPK5-3*, *MAPK6-2*, *MAPK13-1*, *MAPK16-4*, and *MAPK20-2* as compared to the pRAP15-*ccd*B control (Niraula et al. 2020b). These genes have been further compared to syncytium-expressed genes identified from two different *H. glycines*-resistant genotypes undergoing their defense responses (Niraula et al. 2020b). The cross comparison of these *MAPK*-OE and syncytium-expressed gene lists results in the identification of 8 putatively secreted proteins occurring in common between these studies having a defense function (Niraula et al. 2020b).

The GO analyses generate much larger lists of genes showing their understood biological role(s). These genes can be used in functional transgenic studies as done by Niraula et al. (2020b) to determine whether they exhibit a defense role. The *MAPK3-1*-OE and *MAPK3-2*-OE lists have 41.3% and 37.6% induced genes in common, respectfully. Analyses presented here show the *MAPK3-1* and *MAPK3-2* can induce the expression of genes like several *polygalacturonase inhibiting protein 1* (*PGIP1*) paralogs (Glyma.05G123700, Glyma.08G078900, Glyma.05G123900), and the *AtPEPR1* PRR receptor (Glyma.10G195700) that function in defense (Liu et al. 2013; Li and Smigocki 2018; Jing et al. 2020). In *A. thaliana* PEPR1 recognizes short peptides, leading to the activation of BRI-ASSOCIATED KINASE 1 (BAK1) and BOTRYTIS INDUCED KINASE1 (BIK1) to promote defense responses through MAPK signaling. In *Pennisetum glaucum* (pearl millet), its *MAPK4* (*PgMPK4*) gene functions to induce the expression of the *PGIP* defense gene (Melvin et al. 2015). A *B. vulgaris* (sugar beet) *PGIP* expressed in *Nicotiana benthamiana* (tobacco) functions effectively in limiting the pathogenicity of *Rhizoctonia solani*, *Fusarium solani,* and *Botrytis cinerea* driven by their polygalacturonases (PGs) (Li and Smigocki 2018). The results presented here provide confidence that transgenic expression of the *G. max MAPK*s in *G. hirsutum* may function effectively in driving a defense response to *M. incognita* parasitism.

### *MAPK3-1* and *MAPK3-2* can be expressed in *G. hirsutum*

The functional genetic engineering experiments succeeded in generating transgenic roots in *G. hirsutum* that are heterologously expressing the *MAPK3-1* and *MAPK3-2* genes. The ability to obtain transgenic roots that are expressing the target gene is not guaranteed. Austin et al. (2019) examined a family of syncytium-expressed myosin XI genes including Glyma.06G056500, Glyma.13G281900, Glyma.17G051900, Glyma.19G170700, and Glyma.20G001300, targeting them for overexpression in *G. max*. In *A. thaliana*, myosin XI functions in plant defense in processes involving vesicle transport and callose deposition (Yang et al. 2014). The overexpression of the myosin XI genes never led to the production of transgenic roots while control pRAP15-*ccd*B roots and roots targeted for overexpression of other genes could be obtained (Austin et al. 2019). In contrast, RNAi for the targeted myosin genes did generate roots with suppressed expression of the targeted gene and an increase in *H. glycines* parasitism while altering callose deposition (Austin et al. 2019The production of transgenic *G. hirsutum* expressing the *MAPK3-1* and *MAPK3-2* are produced successfully here, examining their potential role in defense to *M. incognita*.

### *MAPK3-1* and *MAPK3-2* expression in *G. hirsutum* suppresses *M. incognita* gall production

The expression of *MAPK3-1* leads to an 80.32% reduction in the production of galls in analyses of the whole root system, while there is a 68.11% reduction in the production of galls in analyses of the galls per gram of root system. The expression of *MAPK3-2* leads to an 73.46% reduction of the production of galls in analyses of the whole root system, while there is a 66.88% reduction of the production of galls in analyses of the galls per gram of root system. MAPK3 functions downstream of NDR1 and harpin in ETI, making comparisons to our prior experiments expressing *NDR1-1* in *G. hirsutum* relevant (Desikan et al. 1999; Knepper et al. 2011; Lang et al. 2021). Our prior results show the expression of *NDR1-1* in *G. hirsutum* leads to a 70.7% reduction in gall production in whole root system analyses, while a 71.03% reduction in galls per gram of root system is observed (McNeece et al. 2017). However, visibly obvious galls are not a prerequisite for *M. incognita* parasitism in some plants, so it is not an ideal marker for factors that negatively impact *M. incognita* parasitism..

### *MAPK3-1* and *MAPK3-2* expression in *G. hirsutum* suppresses *M. incognita* egg mass production

A more direct way to determine the outcome of the expression of *MAPK3-1* and *MAPK3-2* on *M. incognita* is to examine their effect on egg mass production. The expression of *MAPK3-1* leads to an 82.37% reduction in the production of egg masses in analyses of the whole root system, while there is a 68.78% reduction in the production of egg masses in analyses per gram of root system. The expression of *MAPK3-2* leads to a 76.79% reduction in the production of egg masses in analyses of the whole root system, while there is a 69.69% reduction in the production of egg masses in analyses per gram of root system. The expression of *G. max NDR1-1* in *G. hirsutum* leads to a 53% reduction in egg mass production in whole root system analyses, while a 58.27% reduction in egg masses per gram of root system are observed (McNeece et al. 2017). Therefore, the heterologous expression of *MAPK3-1* or *MAPK3-2* individually is more effective in generating a defense response than *NDR1-1* is on its own. The results indicate that signals from more than one signaling pathway, perhaps PTI, converge on MAPK3 (Asai et al. 2002; Desikan et al. 2002; McNeece et al. 2019). In *G. max*, the overexpression of PTI components functioning upstream of MAPK3, including *BAK1-1* and *BIK1-6*, along with the ETI component *NDR1* all induce *MAPK3* expression, leads to defense gene expression (Pant et al. 2014; McNeece et al. 2017, 2019; Klink et al. 2021a).

### *MAPK3-1* and *MAPK3-2* expression does not suppress *M. incognita* egg production

The expression of *MAPK3-1* leads to a 1.29-fold increase in egg production in analyses of the whole root system, while there is a 2.27-fold increase in egg production in analyses of the eggs per gram of root system. Similarly, the expression of *MAPK3-2* leads to a 1.57-fold increase in the production of egg masses in analyses of the whole root system, while there is a twofold increase in the production of egg masses in analyses per gram of root system. The expression of *G. max NDR1-1* in *G. hirsutum* leads to a 66.9% reduction in egg production in whole root system analyses, while a 73% reduction in eggs per gram of root system is observed (McNeece et al. 2017). These observations lie in contrast to the results obtained for both *MAPK3-1* and *MAPK3-2* expression in *G. hirsutum* where an increase in eggs is observed. The results indicate that there may be aspects of *G. hirsutum* gene signaling pathways that lie upstream of MAPK3-1 and MAPK3-2, also negatively impacting *M. incognita* egg production, that are not engaged by the expression of *MAPK3* which functions downstream of NDR1 (Qin et al. 2018). Alternatively, other functions, including downstream processes are involved (Li et al. 2016).

### *MAPK3-1* and *MAPK3-2* expression suppresses *M. incognita* J2 production

Experiments show that the expression of *G. max MAPK3-1* leads to an 88.21% reduction in the production of J2s in analyses of the whole root system, while there is a 76.98% reduction of the production of J2s in analyses per gram of root system. The expression of *G. max MAPK3-2* leads to an 84.07% reduction of the production of J2s in analyses of the whole root system, while there is a 66.88% reduction of the production of J2s in analyses per gram of root system. In comparison to our prior results, the expression of *G. max NDR1-1* in *G. hirsutum* leads to a 60.67% reduction in J2 production in whole root system analyses, while a 66.57% reduction in eggs per gram of root system is observed (McNeece et al. 2017). These observations made for the expression of *G. max NDR1-1* in *G. hirsutum* exhibits similarity with the results obtained for both *MAPK3-1* and *MAPK3-2* expression in *G. hirsutum*.

### *MAPK3-1* and *MAPK3-2* expression in *G. hirsutum* decreases the *M. incognita* reproductive factor

**Fig. 10:**
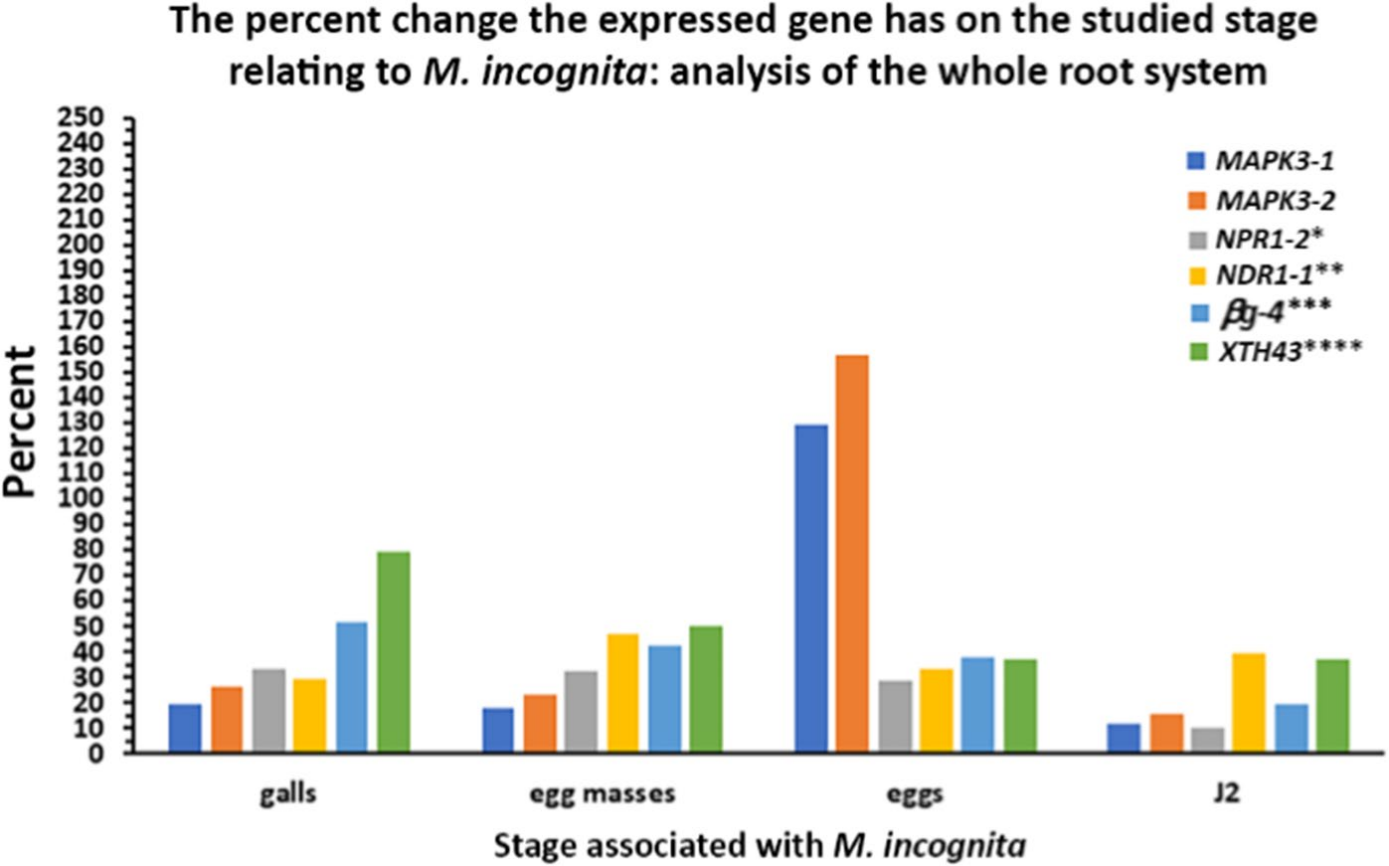
Comparison of the percent effect that *G. max MAPK3-1* or *MAPK3-2* expression has on *M. incognita* gall, egg mass, eggs and J2 production to prior analyses of *NPR1-2*, *NDR1-1*, *g-4*, and *XTH43*. *, ** *NPR1-2*, *g-4* (Pant et al. 2016), *** *NDR1-1* (McNeece et al. 2017), **** *XTH43* (Niraula et al. 2020a)

MAPK3 functions as a signaling node, receiving input from PTI and ETI defense branches (Desikan et al. 1999; Yi et al. 2015; McNeece et al. 2019; Yuan et al. 2021; Lang et al. 2021). Experiments presented here demonstrate a significant, negative effect that the expression of the 2 *MAPK3* genes have on *M. incognita* gall, egg mass, and J2 production in *G. hirsutum* (Fig. 10). Prior experiments examining whether the expression of genes exert their effects on *M. incognita* development in *G. hirsutum* have examined the PTI-regulated *NPR1* (*G. max NPR1-2*), ETI regulated *NDR1* (*G. max NDR1-1*), and two secreted genes including a *g* and *XTH* (*G. max g-4* and *XTH43* (Fig. 10). The *G. max NDR1-2* overexpression in *G. max* increases the relative transcript abundance of soybean *NPR1-2*, *g-4*, and *XTH43* (McNeece et al. 2017).

The results for the *M. incognita*-induced gall studies show that the *MAPK3-1*-E (68.11% reduction), and *MAPK3-2*-E (66.88% reduction) root systems are about as effective as the *G. max NDR1-1*-E (71.03% reduction), and *NPR1-2*-E (66.01% reduction) root systems in decreasing gall number (Fig. 10). However, the expression of the *G. max* secreted *g-4* (56.97% reduction), and *XTH43* (17.7% reduction) are less effective. The similarity in outcome of *MAPK3-1* and *MAPK3-2* to those obtained for *NDR1-1* and *NPR1-2* are consistent with the proteins functioning in the same genetic pathway. Harpin signals through NDR1 and MAPK, leading to a defense response (Gopalan et al. 1996; Desikan et al. 1999, 2001; Lee et al. 2001; McNeece et al. 2019; Lang et al. 2021). In *G. max*, *NDR1-1* overexpression leads to the induced expression of *MAPK3-2* (which leads to the induced expression of *RO-40*, *TGA2-1*, *SHMT-5*, and *NPR1-1*), *MAPK20-2* (which leads to the induced expression of *TGA2-1*, *EDS1-1*, *RO-40*, *GS-3*, *MAMMALIAN UNCOORDINATED* (*MUNC*), and *PR1-6*) (Falk et al. 1999; McNeece et al. 2019). In *G. max*, the *NPR1-2* overexpression cassette induces its own expression (*NPR1-2*) while also increasing the relative transcript abundances of *XTH43*, *BIK1-6*, the salicylic acid regulated secreted protein gene *PR1-6*, the ethylene and jasmonic acid responsive secreted protein gene basic chitinase *PR3* (Glyma.02G042500), the *rhg1* locus component *amino acid transporter* (*AAT*) (Glyma.18G022400), and *SHMT-5* (Antoniw and Pierpoint 1978; Legrand et al. 1987; Liu et al. 2012; Pant et al. 2014).. In these analyses *MAPK3-1*-E and *MAPK3-2*-E engineered in *G. hirsutum* suppresses *M. incognita* egg mass production by 68.78% and 66.88%, respectively (Fig. 10). *NDR1-1*-E engineered in *G. hirsutum* suppresses egg mass production by 58.27%, while *NPR1-2*-E engineered in *G. hirsutum* suppresses *M. incognita* development by 68.18%, levels similar as *MAPK3-1*-E and *MAPK3-2*-E. The secreted *g-4*-E engineered in *G. hirsutum* suppresses *M. incognita* by 77.4%, while *XTH43*-E engineered in *G. hirsutum* suppresses *M. incognita* by 70%. Generally, each of these genes are functioning at levels that are similar, with highly effective suppression of egg mass production occurring. The disparity between gall production and egg mass formation in *g-4*-E and *XTH43*-E *G. hirsutum* is striking. The results indicate that those genes are not as important for the impairment of gall formation, showing that defense processes targeting gall and syncytium formation do exhibit specificity (Pant et al. 2014; Niraula et al. 2020b). *M. incognita* egg masses are structures that contain the eggs and a gelatinous matrix. A surprise that came out of the analysis of egg quantity is that while *MAPK3-1* and *MAPK3-2* expression leads to a significant decrease (> 66%) in egg mass production, the number of eggs increases significantly by 2.27-fold and twofold, respectively. In contrast, *NDR1-1*, and *NPR1-2* expression in *G. hirsutum* roots decreases egg production by 73% and 77.55%, respectively. The experimentally-expressed *g-4* and *XTH43* in *G. hirsutum* is also highly effective in suppressing *M. incognita* egg production, leading to a 77.55% and 79.55% decrease, respectively. It is unclear why there would be such a significant increase in the amounts of eggs in the *MAPK3-1* and *MAPK3-2*-expressing *G. hirsutum* roots. In a process known as hormesis, altered hormone concentrations in the dipteran insect *Bactrocera dorsalis* occurring by exposure to a low, sublethal concentration of a synthetic anthranilic diamide ryanodine receptor agonist insecticide cyantraniliprole leads to an increase in egg production (Zhang et al. 2015). Furthermore, sublethal doses of plant secondary metabolites can also increase insect fecundity so the effects are not limited to synthetic, non-plant compounds (Papanastasiou et al. 2017). Similar results have been observed in nematodes (Martel et al. 2020). The results presented here indicate that a very specific process is occurring in *G. hirsutum* due to the altered transcriptional program caused by the *MAPK3-1* and *MAPK3-2* expression. Their expression in *G. hirsutum* is leading to the production or absence of a bioactive molecule(s) that produce contrasting impacts, both negative (decreased gall, egg mass, and J2s) and positive (increased egg production) on *M. incognita*. While an increase in *M. incognita* egg production is observed, it is possible that the eggs are inviable and that a mechanism by which *M. incognita* produces viable J2s is impaired in *G. hirsutum* roots expressing *MAPK3-1* and *MAPK3-2*. Having the expression of each *MAPK3* paralog leading to the same outcome across multiple replicates argues against the result being nonspecific. To examine this observation further, an analysis of J2s is performed.

The analysis of *M. incognita* J2 quantity results in the demonstration that the expression of *MAPK3-1* and *MAPK3-2* in *G. hirsutum* leads to a 76.98% and 66.57% decrease, respectively. The expression of *NDR1-1* and *NPR1-2* in *G. hirsutum* leads to a 66.57% and 88.58% decrease in J2s, respectively, while the experimentally-induced *g-4* and *XTH43* gene expression in *G. hirsutum* reduces *M. incognita* J2s by 91.65% and 96.8%, respectfully. What the results indicate for *MAPK3-1* and *MAPK3-2*-expressing *G. hirsutum* roots is that a significant amount of *M. incognita* eggs could be non-viable. In contrast, the *NDR1-1* and *NPR1-2*-expressing *G. hirsutum* roots have J2 values similar with what is observed for galls (71.03% and 66.01% reduction, respectively), egg masses (58.27% and 68.18% reduction, respectively), and eggs (73% and 77.55% reduction, respectively). These results indicate that the effects exerted on *M. incognita* by *G. max NDR1-1* and *NPR1-2* expression in *G. hirsutum* occur early during infection and remain in place throughout the plant-nematode interaction during gall, egg mass, egg, and J2 production. The gene expression results for the *G. max g-4* and *XTH43* differ in that there appears to be a cumulative negative effect that their expression in *G. hirsutum* has on *M. incognita* (Fig. 10). A cumulative negative effect on pathogen fitness is observed for transgenic plants expressing-glucosidases (Zagrobelny et al. 2007, 2008). The trends seen for the effects generated by heterologous gene expression for the examined genes in relation to *M. incognita* galls, egg masses, eggs, and J2s demonstrate that it is possible to target specific aspects of the pathogen life cycle (Scheideler et al. 2002). However, the expression of multiple genes may aid in producing an additive effect that could eliminate infection or parasitism altogether (Zhang and Shapiro 2002; Zhou et al. 2020).

The results presented here show both *G. max g-4* and *XTH43* expression function highly effectively in mitigating *M. incognita* as demonstrated by their 91.65 and 96.8% reduction in J2 production, respectively (Pant et al. 2016; Niraula et al. 2020b). These results are consistent with the effect that *MAPK*-induced, syncytium-expressed secreted proteins have on *H. glycines* development in *G. max* (Niraula et al. 2020b). It is clear from these observations that it is possible to obtain a very high suppression of *M. incognita* development through the expression of genes from heterologous sources. Using promoters that effectively drive that expression in the presence of the nematode which has a significant capacity to control root cell gene expression is an important consideration when studying how genes function during pathogenesis and defense (Klink et al. 2009, 2021a; Ali and Kim 2019). The analysis presented here describes the effect that the *MAPK3-1* and *MAPK3-2* expression has on *M. incognita* parasitism in *G. hirsutum* and discussing those data in relation to the previously studied *NDR1-1*, *NPR1-2*, *g-4*, and *XTH43* (Pant et al. 2016; McNeece et al. 2017; Niraula et al. 2020b). A comparison of the results obtained for those genes demonstrates that, unlike the *G. max MAPK3-1*, *MAPK3-2*, *NDR1-1*, and *NPR-1-2* signaling genes, the heterologous expression of *g-4* and *XTH43* secreted protein genes clearly have a cumulative negative effect on *M. incognita* at later stages of its life cycle, in particular J2s (Fig. 10). The results show that there are genes functioning effectively at different stages of the defense response that require further exploration as to what their role(s) are. For example, cell wall biochemical analyses show the *G. max XTH43* overexpression shortens XyG chain length, increases the number of those shorter XyG chains and increases the amount of XyG, consistent with earlier cytological and ultrastructural studies of syncytia undergoing a defense response (Ross 1958; Endo 1965, 1991; Niraula et al. 2021). *XTH43*-RNAi has the opposite effect (Niraula et al. 2021). The increase in the number of shorter chains may interfere with a secreted pathogen effector’s ability to enzymatically degrade cell walls by producing a diffusion barrier (Niraula et al. 2020b). Alternatively, the nematode does not produce enough wall-degrading enzyme to combat the extra wall material it encounters.

### *MAPK3-1* and *MAPK3-2* expression in *G. hirsutum* affects its root growth

Differences are observed in the relative amount of *M. incognita*-induced galls, egg masses, eggs, and J2s in analyses of data obtained from *MAPK3-1*-E and *MAPK3-2*-E whole root systems as compared to the numbers per gram of root system, in relation to the pRAP15-*ccd*B control roots. Therefore, the expression of the *MAPK3-1* or *MAPK3-2* transgenes in *G. hirsutum* affects root mass as compared to the respective controls, in these cases negatively. The expression of the *G. max XTH43*, *NPR1-2* or *g-4* in *G. hirsutum* leads to no statistically significant effect on root mass (Pant et al. 2016; Niraula et al. 2020a). XTH43 and g-4, as secreted proteins would be expected to function downstream of MAPK signaling. NPR1-2 functions as a co-transcriptional regulator with TGA2-1 and would be expected to function downstream of MAPK3. Therefore, targeting downstream genes that encode the secreted XTH43 and g-4 may be an effective way to generate resistance while not experiencing the drag of reduced root mass.

### Targeting of MAPK signaling in understudied, but agriculturally relevant crops, for defense

Significant agricultural plant species are presented in recent studies, (Tilman et al. 2011; Ray et al. 2013, 2019). Relating to climate change (). While MAPKs are studied under certain circumstances, in some crops, they are not annotated. The analysis presented here in those cases identify their MAPKs to aid in the analysis of these genes in relation to plant defense and other basic biological roles including climate change (Burkhead and Klink 2018; Li and Smigocki 2018). ().

### Model

**Fig. 11:**
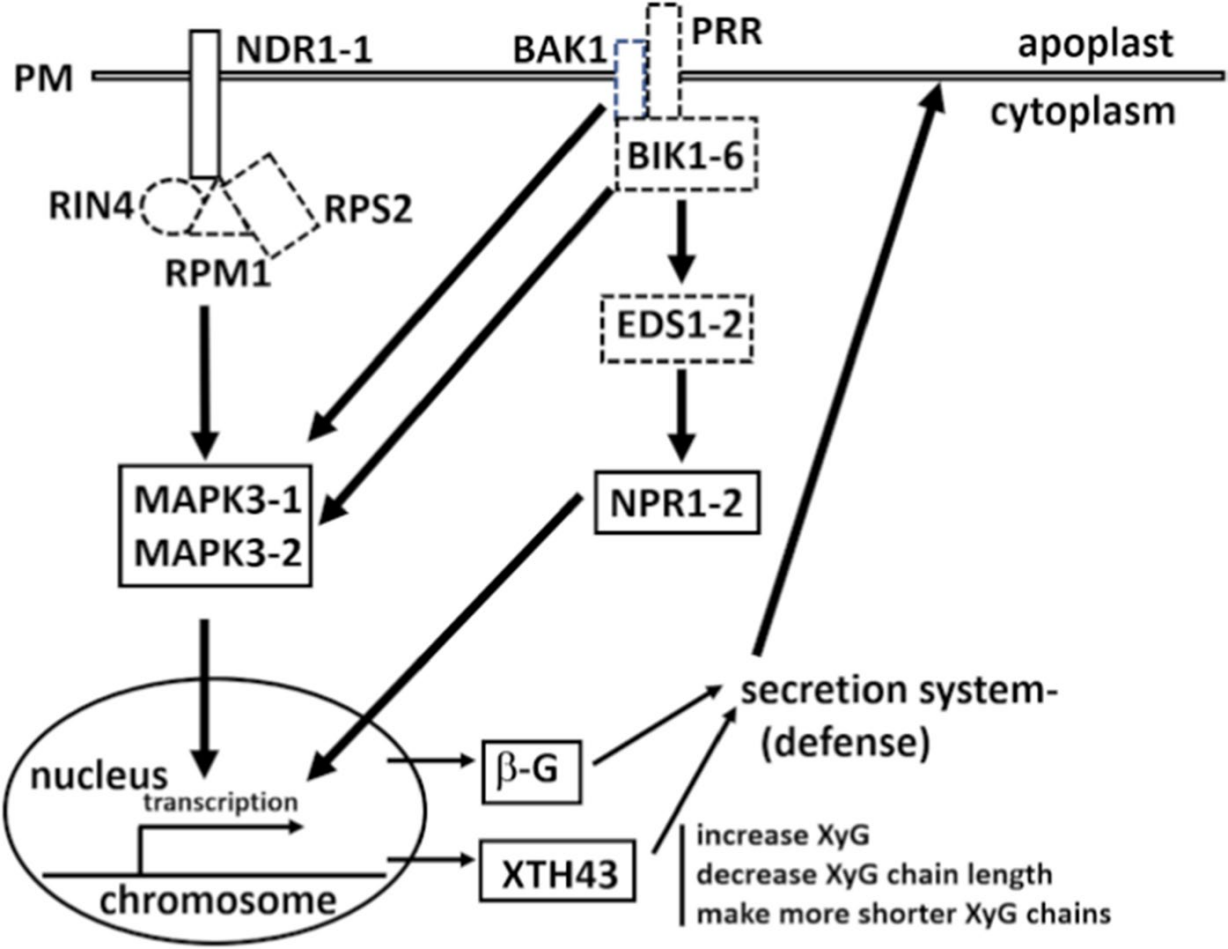
Model. *G. max* ETI and PTI genes expressed in *G. hirsutum* lead to a defense response. These functioning genes include the ETI gene *NDR1* which acts with *RIN4*, *RPM1*, and *RPS2*, whose expression leads to *MAPK* expression and downstream gene expression including the expression of the secreted genes *g-4*, and *XTH43*. PTI genes including PRRs, the co-receptor *BAK1*, the associated cytoplasmic kinase *BIK1* lead to induced *EDS1* and *NPR1* expression. PTI and ETI cross communicate. *EDS1* signals through *NPR1* to activate the transcription of downstream genes functioning in defense. *G. max* genes denoted with hashed lines (i.e., *EDS1*, *BIK1*, *RIN4*, *RPM1*, *RPS2*) have not been heterologously expressed in *G. hirsutum*. Genes with solid lines (i.e., *MAPK3-1*, *MAPK3-2*, *NPR1-2*, *g-4*, *XTH43*) have been heterologously expressed in *G. hirsutum* (Yi et al. 2015; Pant et al. 2016; McNeece et al. 2017; Chen et al. 2017; Liu et al. 2020; Yuan et al. 2021; Niraula et al. 2020a, b)

The experiments presented here show it is possible to genetically engineer in an important node (MAPK) functioning in plant defense signaling acting downstream of the PTI and ETI receptors. The effort has resulted in the generation of a highly resistant reaction,impairing *M. incognita* development (Fig. 11) (Yi et al. 2015; Chen et al. 2017; Niu et al. 2016; Nie et al. 2017; McNeece et al. 2017; Aljaafri et al. 2017).

## Supplementary Information

Below is the link to the electronic supplementary material.

Supplementary file1 (DOCX 816 kb)

## Data Availability

All data is available in this work.
