# Supplementary material for: The heterologous expression of conserved Glycine max (soybean) mitogen activated protein kinase 3 (MAPK3) paralogs suppresses Meloidogyne incognita parasitism in Gossypium hirsutum (upland cotton)
Source: Transgenic Res. 2022 Jun 28;31(4-5):457–87. doi: 10.1007/s11248-022-00312-y (PMC9489592; doi:10.1007/s11248-022-00312-y)
Supplement: Supplementary file 1 — Supplementary file1 (DOCX 816 kb) [file 11248_2022_312_MOESM1_ESM.docx]

**SUPPLEMENTAL FIGURES**

MAPK3-1 1 MAGVNPNGVADFPATPTHGGQFIQYNIFGNLFEVTAKYRPPIMPVGRGAY 50

||||||||||||.|.||||||||||||||||||||.||||||||:|||||

MAPK3-2 1 MAGVNPNGVADFAAVPTHGGQFIQYNIFGNLFEVTTKYRPPIMPIGRGAY 50

MAPK3-1 51 GIVCSLLNTETNELVAVKKIANAFDNHMDAKRTLREIKLLRHLDHENVIG 100

||||||||||||||||||||||||||||||||||||||||||||||||||

MAPK3-2 51 GIVCSLLNTETNELVAVKKIANAFDNHMDAKRTLREIKLLRHLDHENVIG 100

MAPK3-1 101 LRDVIPPPLRREFNDVYIATELMDTDLHHIIRSNQNLSEEHSQYFLYQIL 150

|||||||||||||||||||||||||||||||||||||||||.||||||||

MAPK3-2 101 LRDVIPPPLRREFNDVYIATELMDTDLHHIIRSNQNLSEEHCQYFLYQIL 150

MAPK3-1 151 RGLKYIHSANVIHRDLKPSNLLLNSNCDLKIIDFGLARPTLESDFMTEYV 200

||||||||||||||||||||||||||||||||||||||||||||||||||

MAPK3-2 151 RGLKYIHSANVIHRDLKPSNLLLNSNCDLKIIDFGLARPTLESDFMTEYV 200

MAPK3-1 201 VTRWYRAPELLLNSSDYTSAIDVWSVGCIFMELMNKKPLFPGKDHVHQMR 250

||||||||||||||||||||||||||||||||||||||||||||||||||

MAPK3-2 201 VTRWYRAPELLLNSSDYTSAIDVWSVGCIFMELMNKKPLFPGKDHVHQMR 250

MAPK3-1 251 LLTELLGTPTEADLGLVKNEDARRYIRQLPQYPRQPLAQVFPHVHPAAID 300

||||||||||||||||||||||||||||||||||||||||||||||||||

MAPK3-2 251 LLTELLGTPTEADLGLVKNEDARRYIRQLPQYPRQPLAQVFPHVHPAAID 300

MAPK3-1 301 LVDKMLTVDPTKRITVEEALAHPYLEKLHDVADEPICMEPFSFDFEQQQL 350

||||||||||||||||||||||||||||||||||||||||||||||||||

MAPK3-2 301 LVDKMLTVDPTKRITVEEALAHPYLEKLHDVADEPICMEPFSFDFEQQQL 350

MAPK3-1 351 DEEQIKEMIYREALALNPEYA 371

|||||||||||||||||||||

MAPK3-2 351 DEEQIKEMIYREALALNPEYA 371

**Supplemental Figure 1.** Pairwise comparison between MAPK3-1 and MAPK3-2 protein sequences are made using the EMBOSS Program Needle, Version 6.6.0 using the Matrix; EBLOSUM62; Gap open, 10.0; Gap extend, 0.5; End Gap Penalty, false; End Gap Open Penalty, 10.0; End Gap Extension Penalty, 0.5. “|” exact match, “:” very similar mismatch meaning the amino acids are very similar in their physiochemical profiles; “.” somewhat similar in their physiochemical profiles. The TEY phosphorylation domain of plant MAPK3 activation loop (A-loop) is highlighted in red.

**Supplemental Figure 2.**

Gohir.D12G233400.1.p ---------MKKEMGS------------TKHSSSEGSSIKGVPTHGGKYVHYNVYGNLFE 39

Gohir.A02G009100.1.p MEGGGPPQAADTEMAE--QPNPQNHQQQPPQMAIGLENIPATLSHGGRFIQYNIFGNIFE 58

Gohir.A03G088300.1.p MEGGGPPQAADTEMAEAAQQQPQHHQQRPPQVAAGLENIPATLSHGGRFIQYNIFGNIFE 60

Gohir.D02G108500.1.p MEGGGPPQAADTEMAEAAQQQPQHHQQHPPQVAAGLENIPATLSHGGRFIQYNIFGNIFE 60

Glyma.U021800.1.p ---------------------------MAGVNPNGVADFPATPTHGGQFIQYNIFGNLFE 33

Glyma.12G073000.1.p ---------------------------MAGVNPNGVADFAAVPTHGGQFIQYNIFGNLFE 33

Gohir.A03G035400.1.p -----------------------MADVAPGNAGGQFGDFPTIHTHGGQFIQYNIFGNLFE 37

Gohir.D05G100500.1.p -----------------------MANVAPGNAGGHFGDFPAFHTYGGQFIQYGIFGNLFE 37

.: ::**::::*.::**:**

Gohir.D12G233400.1.p VSSKYVPPIRPIGRGANGIVCAAVNSETRQEVAIKKIGNAFDNIIDARRTLREIKLLRHM 99

Gohir.A02G009100.1.p VTAKYKPPIMPIGKGAYGIVCSALNSETNEQVALKKIANAFDNKIDAKRTLREIKLLRHM 118

Gohir.A03G088300.1.p VTAKYKPPIMPIGKGAYGIVCSALNSETNEHVALKKIANAFDNKIDAKRTLREIKLLRHM 120

Gohir.D02G108500.1.p VTAKYKPPIMPIGKGAYGIVCSALNSETNEHVALKKIANAFDNKIDAKRTLREIKLLRHM 120

Glyma.U021800.1.p VTAKYRPPIMPVGRGAYGIVCSLLNTETNELVAVKKIANAFDNHMDAKRTLREIKLLRHL 93

Glyma.12G073000.1.p VTTKYRPPIMPIGRGAYGIVCSLLNTETNELVAVKKIANAFDNHMDAKRTLREIKLLRHL 93

Gohir.A03G035400.1.p VTSKYRPPIMPIGRGASGIVCSVLNSETNEMVAVKKIANAFDNHMDAKRTLREIKLLRHL 97

Gohir.D05G100500.1.p ITSKYRPPIMPIGRGAYGIVCSVLNSETNEMVAVKKIANAFDNHMDAKRTLREIKLLRHL 97

:::** *** *:*:** ****: :*:**.: **:***.***** :**:***********:

Gohir.D12G233400.1.p DHENVIAIKDIIRPPKKETFNDVYIVYELMDTDLHHIIRSDQPLTDDHCQYFLYQLLRGL 159

Gohir.A02G009100.1.p DHENVVAIRDIIPPPKRECFNDVYIAYELMDTDLHQIIRSNQALSEEHCQYFLYQILRGL 178

Gohir.A03G088300.1.p DHENVVAIRDIIPPPQRECFNDVYIAYELMDTDLHQIIRSNQALSEEHCQYFLYQILRGL 180

Gohir.D02G108500.1.p DHENVVAIRDIIPPPQRECFNDVYIAYELMDTDLHQIIRSNQALSEEHCQYFLYQILRGL 180

Glyma.U021800.1.p DHENVIGLRDVIPPPLRREFNDVYIATELMDTDLHHIIRSNQNLSEEHSQYFLYQILRGL 153

Glyma.12G073000.1.p DHENVIGLRDVIPPPLRREFNDVYIATELMDTDLHHIIRSNQNLSEEHCQYFLYQILRGL 153

Gohir.A03G035400.1.p DHENVIGIKDVIPPPLRREFTDVYIATELMDTDLHQIIRSNQSLSEEHCQYFLYQILRGL 157

Gohir.D05G100500.1.p DHENVIAIRDVIPPPLRRDFTDVYIALELMDTDLHQIIRSNQSLSEEHCQYFLYQLLRGL 157

*****:.::*:* ** :. *.****. ********:****:* *:::*.******:****

Gohir.D12G233400.1.p KYVHSANVLHRDLKPSSLLLNAKCDLKIGDFGLARTTSETDFMTEYVVTRWYRAPELLLN 219

Gohir.A02G009100.1.p KYIHSANVLHRDLKPSNLLLNANCDLKICDFGLARVTSESDFMTEYVVTRWYRAPELLLN 238

Gohir.A03G088300.1.p KYIHSANVLHRDLKPSNLLLNANCDLKICDFGLARVTSESDFMTEYVVTRWYRPPELLLN 240

Gohir.D02G108500.1.p KYIHSANVLHRDLKPSNLLLNANCDLKICDFGLARVTSESDFMTEYVVTRWYRPPELLLN 240

Glyma.U021800.1.p KYIHSANVIHRDLKPSNLLLNSNCDLKIIDFGLARPTLESDFMTEYVVTRWYRAPELLLN 213

Glyma.12G073000.1.p KYIHSANVIHRDLKPSNLLLNSNCDLKIIDFGLARPTLESDFMTEYVVTRWYRAPELLLN 213

Gohir.A03G035400.1.p KYIHSANVIHRDLKPSNLLLNANCDLKICDFGLARPTAENEFMTEYVVTRWYRAPEILLN 217

Gohir.D05G100500.1.p KYIHSAKVIHRDLKPSNLLLNANCDLKICDFGLARPASENEFMTEYVVTRWYRAPEILLN 217

**:***:*:*******.****::***** ****** : *.:************ **:***

Gohir.D12G233400.1.p CSEYTAAIDMWSVGCIFGEIMTREPLFPGKDYVHQLRLITELIGSPDDASLGFLRSNNAR 279

Gohir.A02G009100.1.p SSDYTAAIDVWSVGCIFMELMDRKPLFPGRDHVHQLRLLIELIGTPSEAELGFL-NANAR 297

Gohir.A03G088300.1.p SSDYTAAIDVWSVGCIFMELMDRKPLFPGRDHVHQLRLLMELIGTPSEAELEFL-NENAK 299

Gohir.D02G108500.1.p SSDYTAAIDVWSVGCIFMELMDRKPLFPGRDHVHQLRLLMELIGTPSEAELEFL-NENAK 299

Glyma.U021800.1.p SSDYTSAIDVWSVGCIFMELMNKKPLFPGKDHVHQMRLLTELLGTPTEADLGLVKNEDAR 273

Glyma.12G073000.1.p SSDYTSAIDVWSVGCIFMELMNKKPLFPGKDHVHQMRLLTELLGTPTEADLGLVKNEDAR 273

Gohir.A03G035400.1.p SSDYTAAIDVWSVGCIFMELMNRKPLFPGKDHVHQMRLLTELLGTPTESDLGFLRNEDAR 277

Gohir.D05G100500.1.p SSDYTAAIDVWSVGCIFMELMNRKPLFPGNDHVHQMRLLTELLGTPTESDLGFLQNEDAR 277

.*:**:***:******* *:* ::*****.*:***:**: **:*:* ::.* :: . :*:

Gohir.D12G233400.1.p RYFRQLPQCRKQQFSARFPNMSPGAVDLLEKMLVFDPNKRITAEEALCHPYLASLHDIND 339

Gohir.A02G009100.1.p RYIQQLPLYHRQSFTEKFPTVHPLAIDLVEKMLTFDPRLRITVEDALAHP---------- 347

Gohir.A03G088300.1.p RYIRQLPLYRRQSFTEKFPNVHPLAIDLVEKMLTFDPRQRITVEDALAHPYLTSLHDISD 359

Gohir.D02G108500.1.p RYIRQLPLYRRESFTEKFPNVHPLAIDLVEKMLTFDPRQRITVEDALAHPYLTSLHDISD 359

Glyma.U021800.1.p RYIRQLPQYPRQPLAQVFPHVHPAAIDLVDKMLTVDPTKRITVEEALAHPYLEKLHDVAD 333

Glyma.12G073000.1.p RYIRQLPQYPRQPLAQVFPHVHPAAIDLVDKMLTVDPTKRITVEEALAHPYLEKLHDVAD 333

Gohir.A03G035400.1.p RYIRQLPAHPRQSLAEVFPHVHPLAIDLIDRMLTFDPTRRITVEEALAHPYLERLHDISD 337

Gohir.D05G100500.1.p RYIRQLPAYPRQQLANVYPHVNRLALDLIDRMLTFDPTRRITVEEALAHPYLERLHDIAD 337

**::*** :: :: :* : *:**:::**..** ***.*:**.**

Gohir.D12G233400.1.p EPVCPRPFSFDFEQSSCTEDHIKELIWRESLQFNPDPVH*- 378

Gohir.A02G009100.1.p ----------------------------------------- 347

Gohir.A03G088300.1.p EPVCMTPFSFDFEQHALTEEQMKELIYREALAFNPEYLQQ* 399

Gohir.D02G108500.1.p EPVCMTPFSFDFEQHALTEEQMKELIYREALAFNPEYLQQ* 399

Glyma.U021800.1.p EPICMEPFSFDFEQQQLDEEQIKEMIYREALALNPEYA*-- 371

Glyma.12G073000.1.p EPICMEPFSFDFEQQQLDEEQIKEMIYREALALNPEYA*-- 371

Gohir.A03G035400.1.p EPVCPEPFSFDFEQQPLGEEQMKDMIYQEALAVNPTYA*-- 375

Gohir.D05G100500.1.p EPVCPEPFNFDFEQQPLGEEQMKDMIYREALALNPDYAR*- 376

**Supplemental Figure 2.** Multiple protein sequence alignment between the *G. max* MAPK3-1 and MAPK3-2 protein sequences and the *G. hirsutum* MAPKs from it’s a and D genomes. The TEY phosphorylation domain of plant MAPK3s activation loop (A-loop) is highlighted in red.

**Supplemental Figure 3.**

**
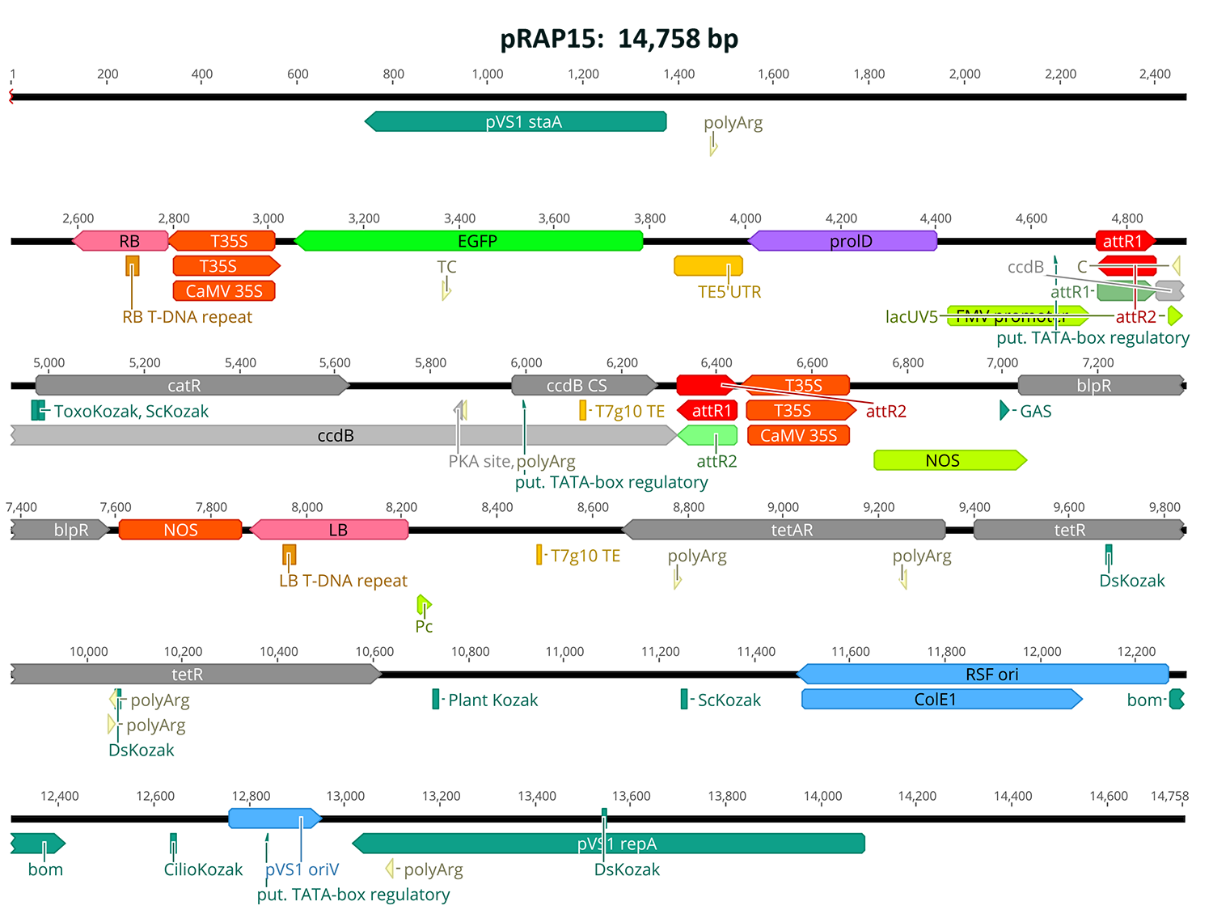
**

**Supplemental Figure 3**. The pRAP15 plasmid.

**Supplemental Figure 4**.


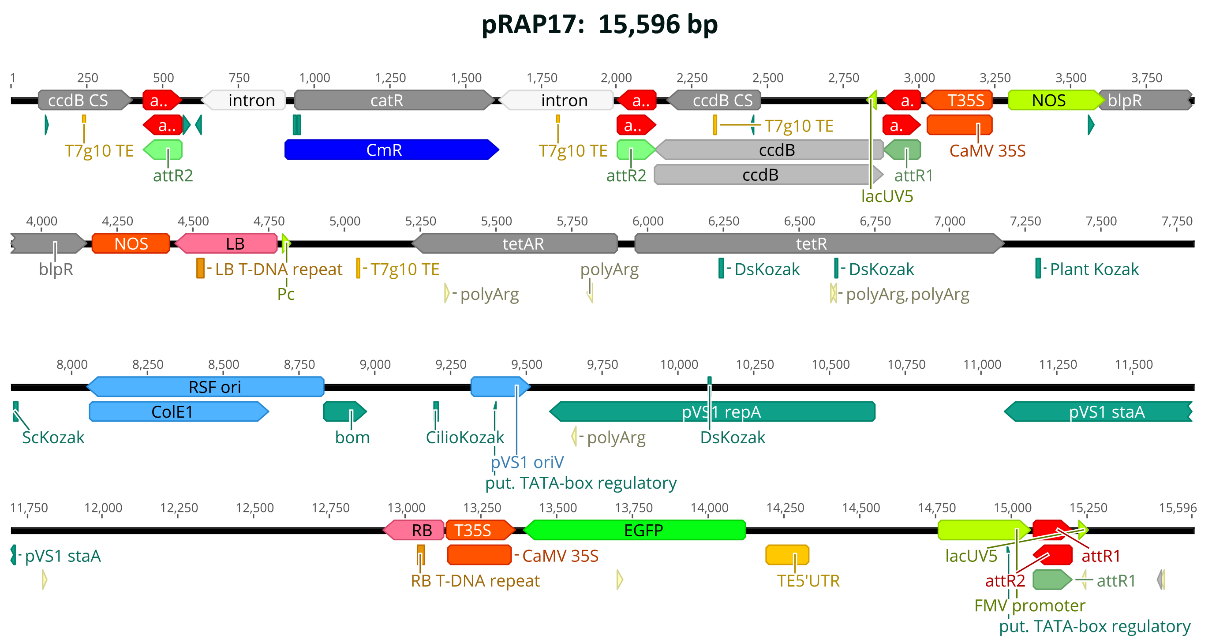


**Supplemental Figure 4**. The pRAP15 plasmid.

**Supplemental Figure 5**.


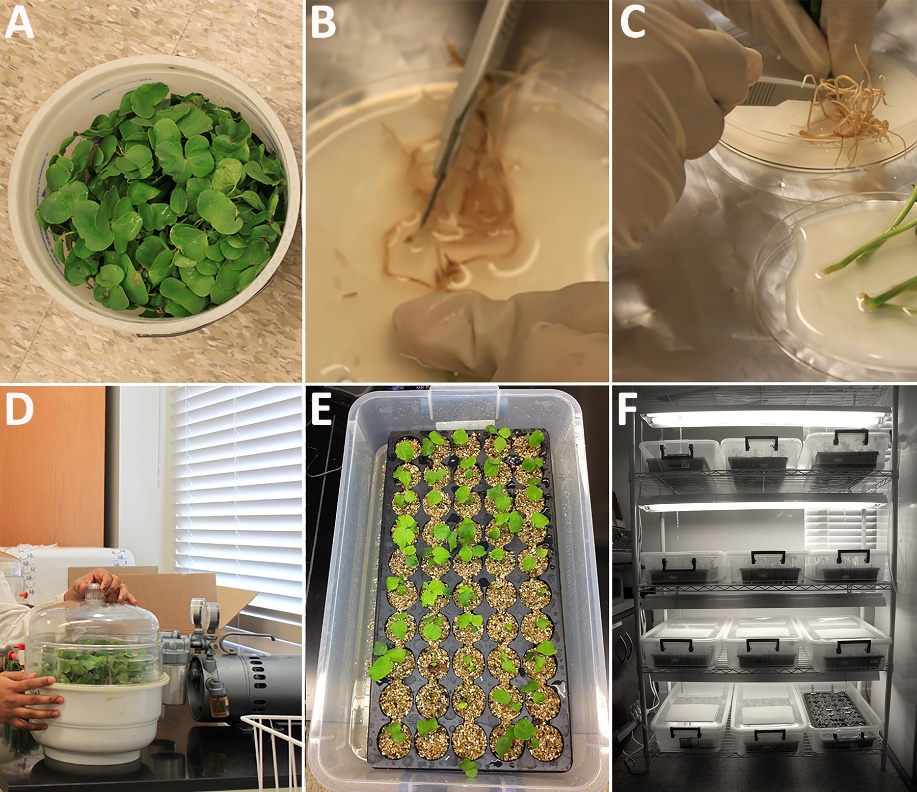


**Supplemental Figure 5**. *G. hirsutum* transgenesis procedure. **A**. Collection of *G. hirsutum* from the greenhouse. **B**. Excision of the *G. hirsutum* root in 15834-containing MS media using a fresh sterile razor blade. **C**. Placing excised plants into 15834-containing MS media. **D**. Placement of root-less *G. hirsutum* in 25 ml of 15834-containing MS media in 140 ml beakers under a vacuum. **E**. Placement of excised hypocotyls into coarse A3 vermiculite in 50-cell trays. **F**. Placement of plants into Sterlite humidity chambers under fluorescent lights. Please refer to Materials and Methods section; subsection, *G. hirsutum* genetic transformation, for details.
